# Supplementary material for: Root exudate metabolites drive plant-soil feedbacks on growth and defense by shaping the rhizosphere microbiota
Source: Nat Commun. 2018 Jul 16;9:2738. doi: 10.1038/s41467-018-05122-7 (PMC6048113; doi:10.1038/s41467-018-05122-7)
Supplement: Supplementary file 1 — Supplementary Information [file 41467_2018_5122_MOESM1_ESM.pdf]

Supplementary Information

Root exudate metabolites drive plant-soil feedbacks on growth and defense by shaping the rhizosphere microbiota

Hu et al.

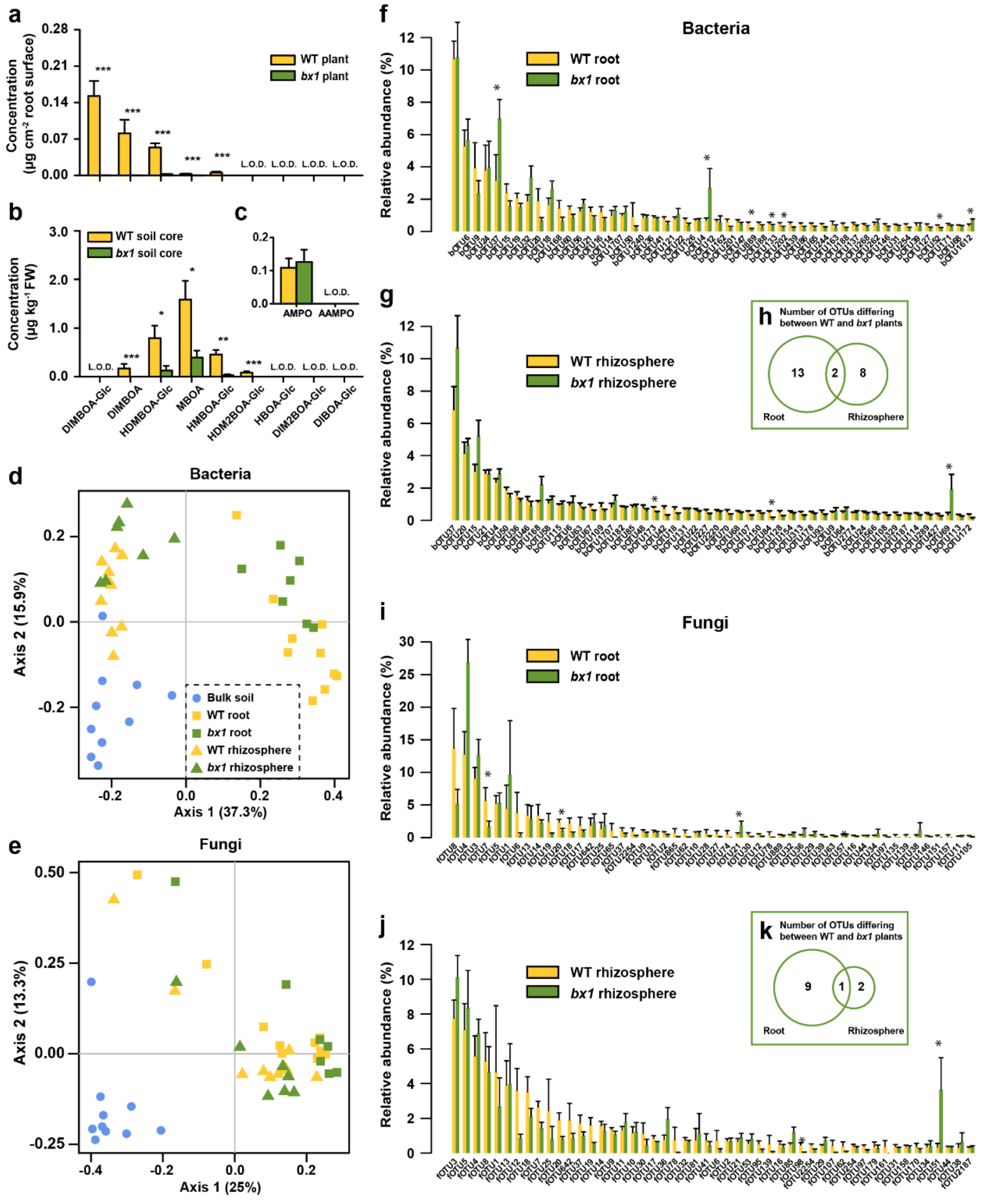

Supplementary Figure 1. Principal Coordinate Analysis of microbiome profiles, and individual microbial operational taxonomic units (OTUs) in the root and rhizosphere from the field experiment. a, Average concentrations of benzoxazinoids (BXs) on the root surface of one week old wild type (WT) B73 and *bx1* mutant maize plants (**a**, +SE, *n*=14-15). **b, c,** Average concentrations of BXs (**b**) and the BX breakdown products AMPO and AAMPO (**c**) in soil cores that were conditioned by wild type (WT) B73 or *bx1* mutant plants for 3 months (+SE, *n*=5). Asterisks indicate significant differences between genotypes (**P* < 0.05; ***P* < 0.01; *** *P*< 0.001, two-sided Student's *t*-tests).**d, e,** Unconstrained ordinations of 16S rRNA bacterial gene profiles (**d**) and fungal internal transcribed spacer profiles (**e**) profiles of soils, rhizosphere and root samples based on Bray Curtis distances. Data points represent individual replicate samples (*n*=7-10). The field

dataset contained 3,107 and 1,861 bacterial OTUs (bOTUs) and fungal OTUs (fOTUs), of which 184 and 118 had a higher minimal average abundance than 0.1%, respectively. The rank abundance bargraphs (**f, g, i, j**) report the top 50 abundant OTUs. **f, g,** Relative abundance of bacterial OTUs in the roots (**f**) and rhizosphere (**g**) of field-grown wild type (WT) B73 and *bx1* mutant maize plants. Asterisks indicate significant differences between genotypes ($P < 0.05$ (FDR); edgeR's likelihood ratio test, +SE, n=7-10). **h,** Venn diagram of bacterial OTUs differing between WT and *bx1* in root and rhizosphere samples. **i, j,** Relative abundance of fungal OTUs in the roots (**i**) and rhizosphere (**j**) of field-grown wild type (WT) B73 and *bx1* mutant maize plants. Asterisks indicate significant differences between genotypes ($P < 0.05$ (FDR); edgeR's likelihood ratio test, +SE, n=7-10). **k,** Venn diagram of fungal OTUs differing between WT and *bx1* in root and rhizosphere samples. L.O.D., below limit of detection. FW, fresh weight. DIMBOA-Glc: (2-(2,4-dihydroxy-7-methoxy-1,4-benzoxazin-3-one)-β-d-glucopyranose), HDMBOA-Glc: (2-(2-hydroxy-4,7-dimethoxy-1,4-benzoxazin-3-one)-β-d-glucopyranose), HMBOA-Glc: (2-(2-hydroxy-7-methoxy-1,4-benzoxazin-3-one)-β-d-glucopyranose), DIM_2BOA-Glc: (2-(2,4-dihydroxy-6,7-dimethoxy-l,4-benzoxazin-3-one)-β-d-glucopyranose), HBOA-Glc: (2-(2-hydroxy-1,4-benzoxazin-3-one)-β-d-glucopyranose), HDM_2BOA-Glc: (2-(2-hydroxy-4,7,8-trimethoxy-1,4-benzoxazin-3-one)-β-d-glucopyranose, DIBOA-Glc: (2-(2,4-dihydroxy-1,4-benzoxazin-3-one)-β-d-glucopyranose, DIMBOA: 2,4-dihydroxy-7-methoxy-1,4-benzoxazin-3-one, MBOA: 6-methoxy-benzoxazolin-2-one. AMPO: 2-amino-7-methoxy-3H-phenoxazin-3-one, AAMPO: 2-acetylamino-7-methoxy-3H-phenoxazin-3-one.

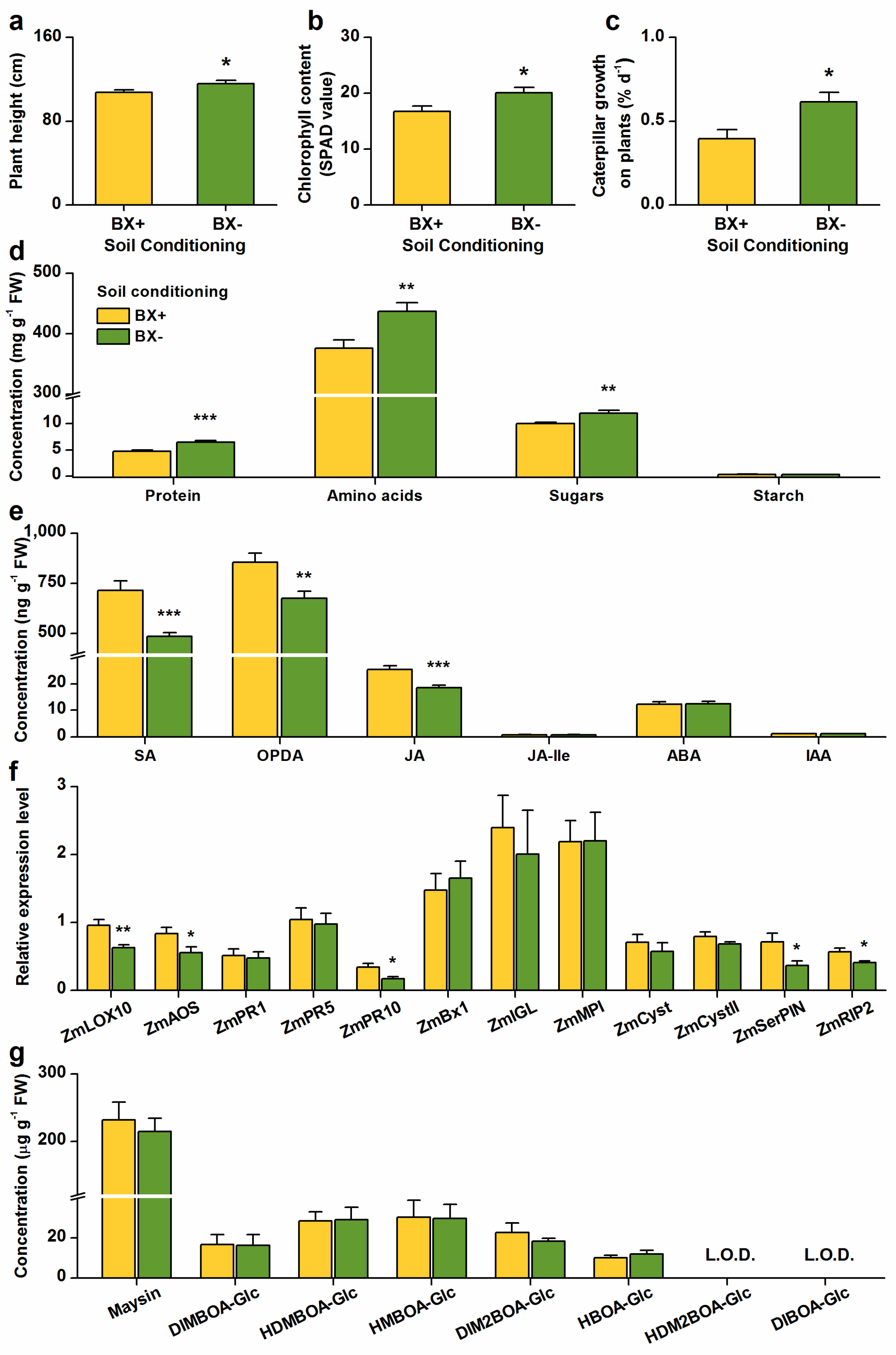

Supplementary Figure 2. Influence of benzoxazinoid (BX) soil conditioning on growth and defense of the next plant generation. a, b, Average plant height (**a**) and chlorophyll contents (**b**) of next generation of wild type (WT) B73 maize plants grown in soils

previously conditioned by WT (BX+) or BX-deficient *bx1* mutant plants (BX-) (+SE, *n*= 10). **c,** Average growth rate of *S. frugiperda* caterpillars feeding on intact WT maize plants grown in BX+ or BX- soils (+SE, *n*=10). **d-g,** Average concentrations of primary metabolites (**d**), leaf phytohormones (**e**), defense marker genes (**f**), and secondary metabolites (**g**) of the next generation of WT maize plants grown in BX+ or BX- soils (+SE, *n*=9-10). FW, fresh weight; L.O.D., below limit of detection; OPDA, 12-oxophytodienoic acid; JA, jasmonic acid; JA-Ile, JA-isoleucine; SA, salicylic acid; IAA, indole-3-acetic acid; ABA, abscisic acid; DIMBOA-Glc, 2-(2,4-dihydroxy-7-methoxy-1,4-benzoxazin-3-one)-β-d-glucopyranose; HDMBOA-Glc, 2-(2-hydroxy-4,7-dimethoxy-1,4-benzoxazin-3-one)-β-d-glucopyranose; HMBOA-Glc, 2-(2-hydroxy-7-methoxy-1,4-benzoxazin-3-one)-β-d-glucopyranose; DIM_2BOA-Glc, 2-(2,4-dihydroxy-6,7-dimethoxy-l,4-benzoxazin-3-one)-β-d-glucopyranose; HBOA-Glc, 2-(2-hydroxy-1,4-benzoxazin-3-one)-β-d-glucopyranose; HDM_2BOA-Glc, 2-(2-hydroxy-4,7,8-trimethoxy-1,4-benzoxazin-3-one)-β-d-glucopyranose; DIBOA-Glc, 2-(2,4-dihydroxy-1,4-benzoxazin-3-one)-β-d-glucopyranose; Asterisks indicate significant differences between soil types (*P < 0.05;**P < 0.01; *** P< 0.001, two-sided Student's *t* tests).

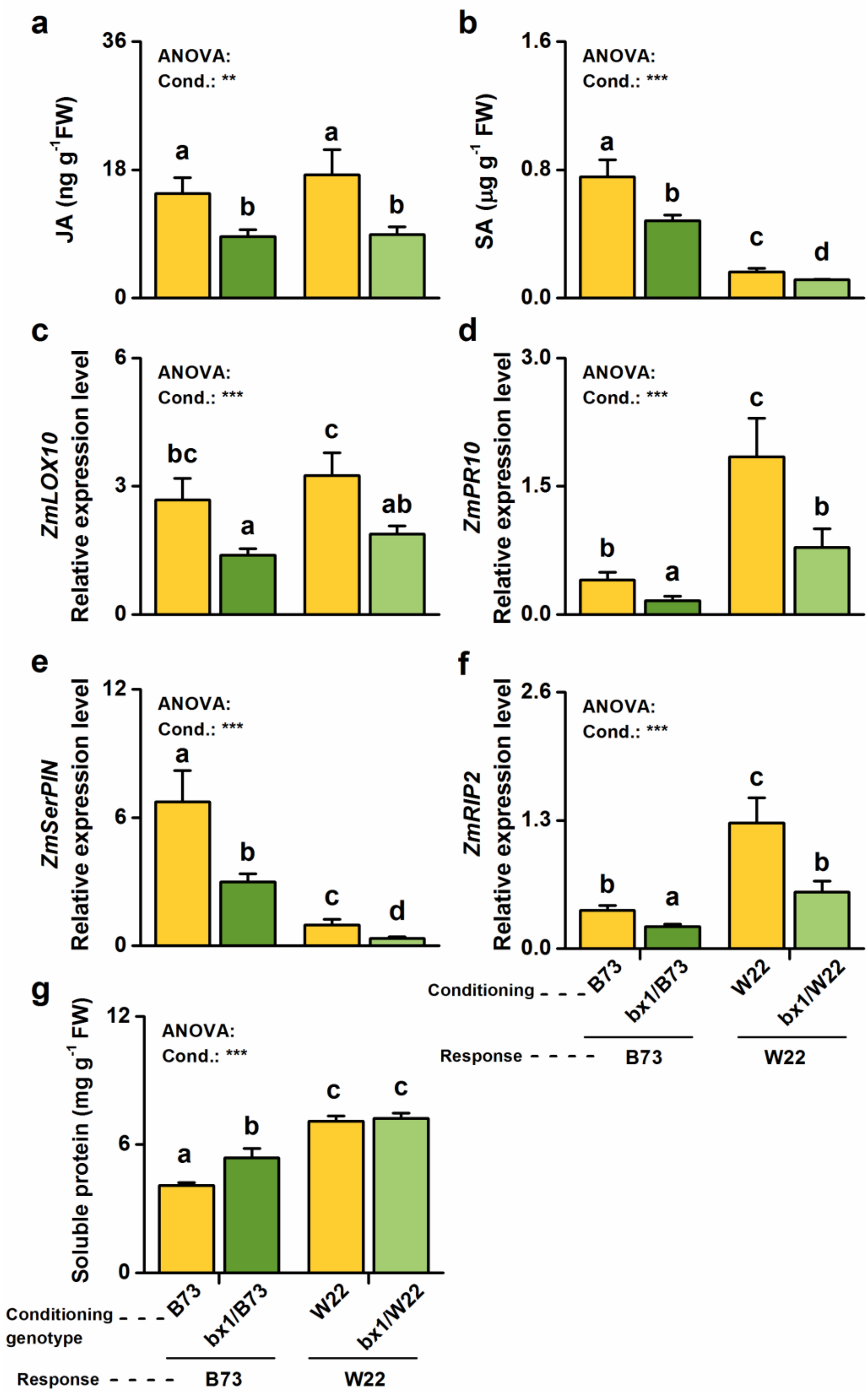

Supplementary Figure 3. Influence of soil conditioning of different benzoxazinoid (BX) mutants on leaf metabolic markers. Average contents of jasmonic acid (JA, **a**), and salicylic acid (SA, **b**); expression levels of *ZmLOX10* (**c**), *ZmPR10* (**d**), *ZmSerPIN* (**e**), and *ZmRIP2* (**f**); concentration of soluble protein (**g**) in the leaves of the next generation of wild type (WT) B73 or W22 maize plants ("Response genotype") growing in soils previously

conditioned by corresponded WT (BX+) or BX-deficient *bx1* mutant plants (BX-) ("Conditioning genotype", +SE, *n*=10). Different letters indicate significant differences between individual soil types (*P* < 0.05, one-way ANOVA followed by multiple comparisons through FDR-corrected LSMeans). FW, fresh weight. Cond., conditioning. Asterisks indicate significant differences between soil types (***P* < 0.01; ****P* < 0.001, one-way ANOVA).

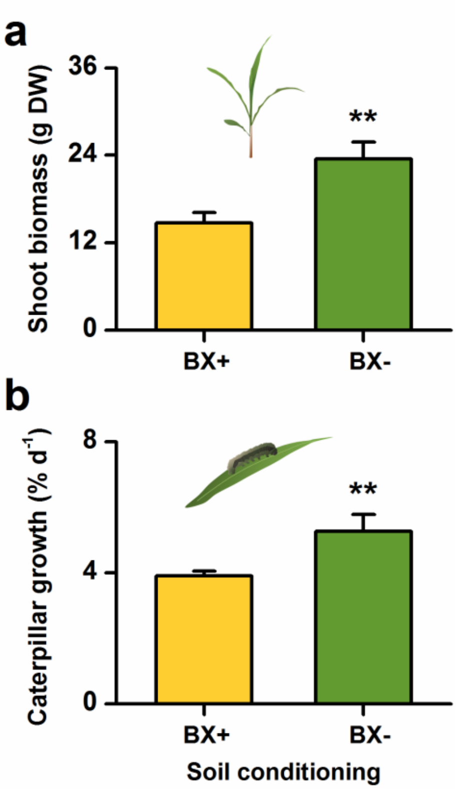

Supplementary Figure 4. Influence of overwintering on plant and herbivore performance. Average shoot biomass (**a**) and caterpillar growth (**b**) of the next generation of B73 maize plants growing in the overwintered soils previously conditioned by wild type (BX+) or BX-deficient *bx1* mutant plants (BX-) (+SE, *n*=11-13). Asterisks indicate significant differences between soil types (***P* < 0.01; two-sided Student's *t* tests). DW, dry weight.

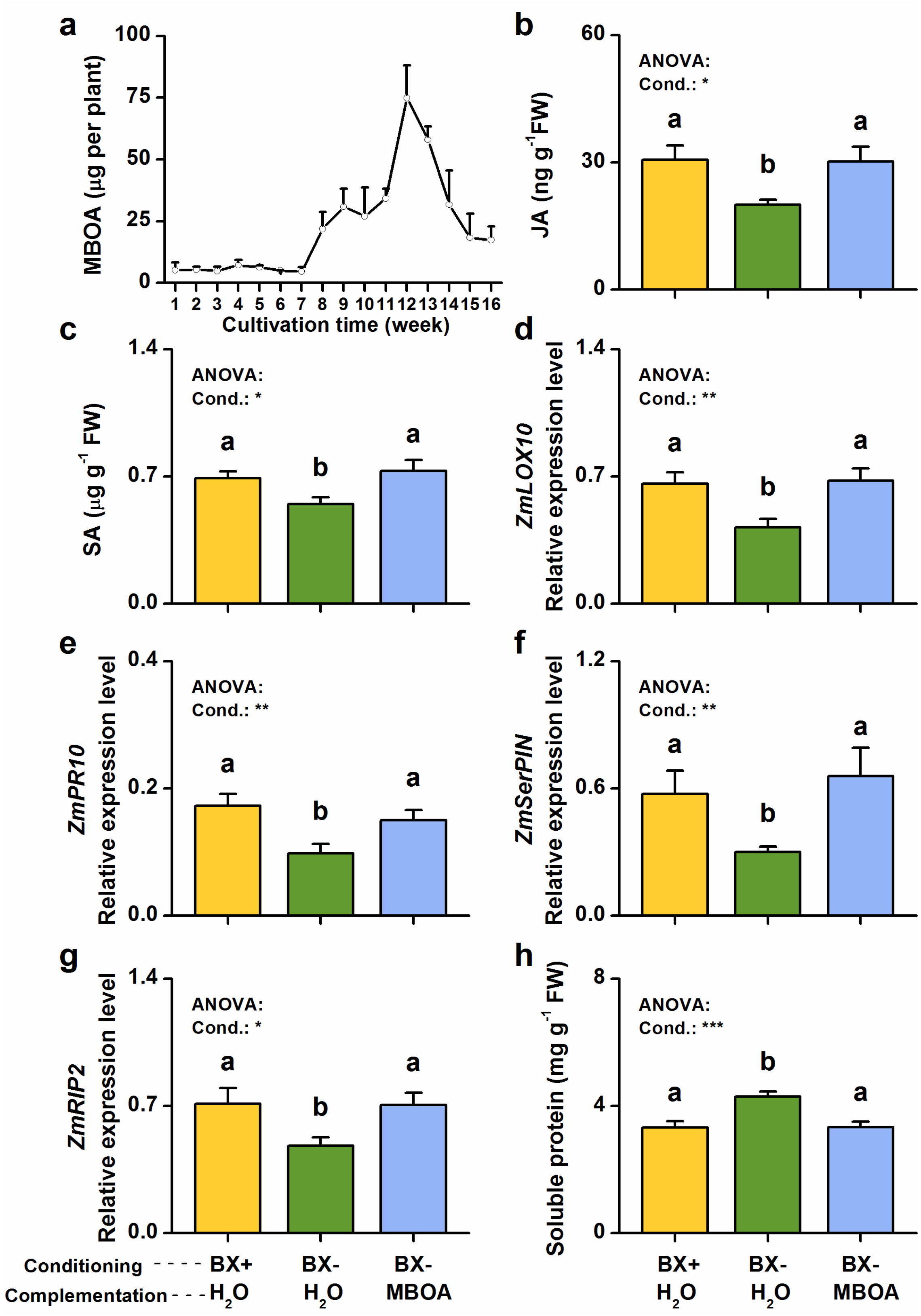

Supplementary Figure 5. Influence of 6-methoxy-benzoxazolin-2-one (MBOA) complementation on leaf metabolic markers. a, Average MBOA amounts recovered from the soils of pot-grown wild type (WT) B73 plants over 16 weeks of cultivation (+SE, *n*=4). Corresponding amounts were applied to the soils of *bx1* mutant plants in the BX

complementation experiment. **b**-**h**, Average contents of jasmonic acid (JA, **b**), and salicylic acid (SA, **c**); expression levels of *ZmLOX10* (**d**), *ZmPR10* (**e**), *ZmSerPIN* (**f**), and *ZmRIP2* (**g**); concentration of soluble protein (**h**) in the leaves of wild type (WT) B73 plants growing in soils previously conditioned by WT (BX+) or BX-deficient *bx1* mutant plants (BX-) ("Conditioning") as well as BX- soils which were supplemented with MBOA during the conditioning phase ("Complementation", +SE, n= 15-19). FW, fresh weight. Cond., conditioning. Asterisks indicate significant differences between soil types (*$P < 0.05$; **$P < 0.01$; ***$P < 0.001$, one-way ANOVA). Different letters indicate significant differences between individual soil types ($P < 0.05$, one-way ANOVA followed by multiple comparisons through FDR-corrected LSMeans).

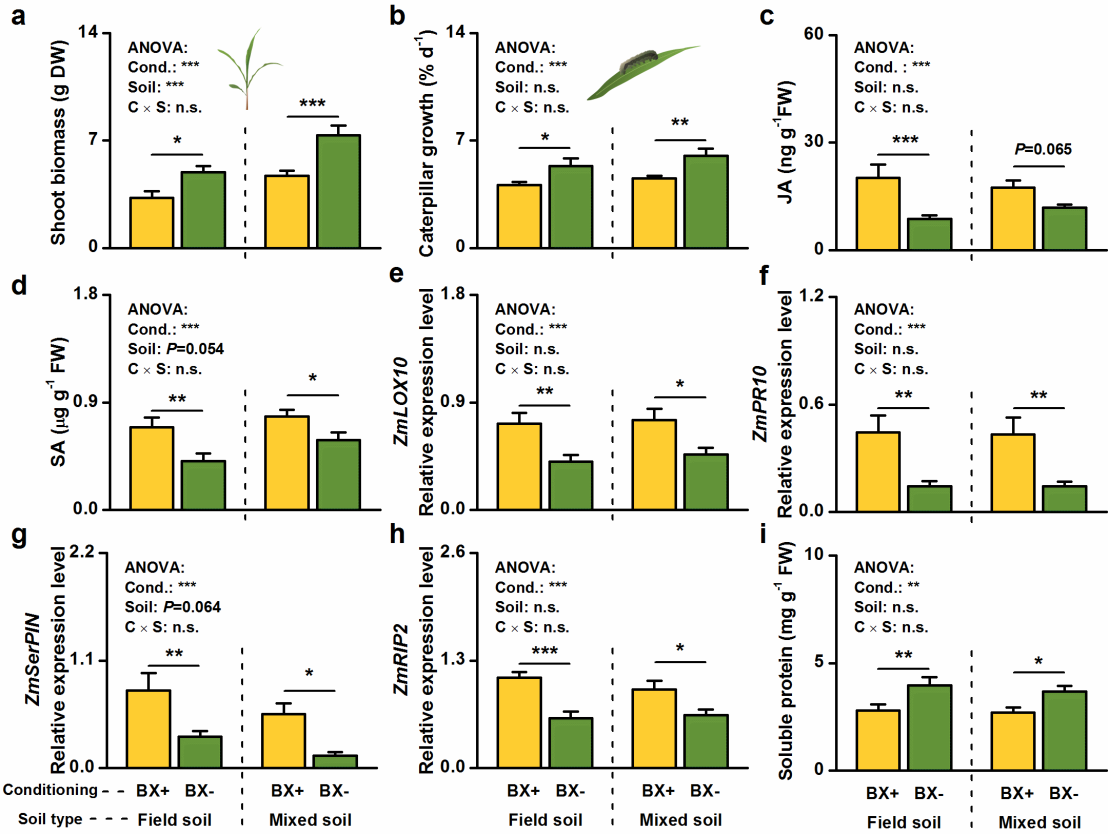

Supplementary Figure 6. Influence of soil structure on benzoxazinoid (BX)-dependent effects. Average shoot biomass (**a**), caterpillar growth (**b**), phytohormone concentrations (**c-d**), defense marker gene expression (**e-h**) and leaf soluble protein (**i**) in wild type (WT) B73 plants growing in soils previously conditioned by WT (BX+) or BX-deficient *bx1* mutant plants (BX-) ("Conditioning") with and without the addition of 50% unconditioned potting soil ("Soil type", +SE, *n*=9-10). Cond. or C, conditioning; S, soil; n.s., no significant; DW, dry weight; FW, fresh weight. The results of two-way ANOVAs are shown. Asterisks indicate significant differences between conditioning treatments within soil types (**P* < 0.05; ***P* < 0.01; ****P*<0.001, two-way ANOVAs followed by paired comparisons through FDR-corrected LSMeans).

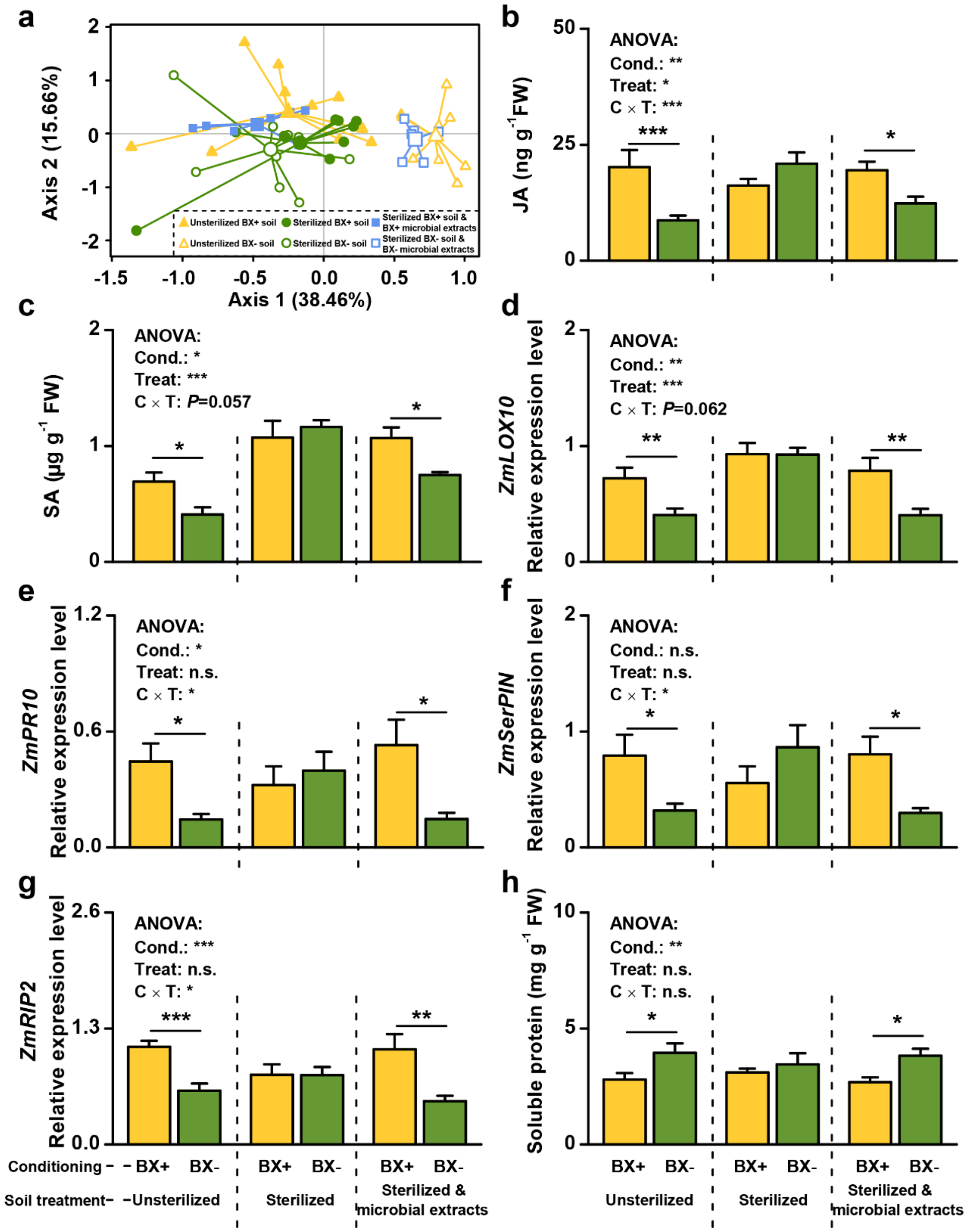

Supplementary Figure 7. Influence of sterilization and microbial complementation on leaf metabolic markers. a, Principal component analysis of leaf markers of wild type (WT) B73 plants growing in soils previously conditioned by WT (BX+) or BX-deficient *bx1* mutant plants (BX-) which were sterilized and supplemented with microbial extracts of BX+ or BX- soils. (*n*=6-10). Data points represent individual replicate samples. **b-h,** Average contents of jasmonic acid (JA, **b**), and salicylic acid (SA, **c**); expression levels of *ZmLOX10* (**d**), *ZmPR10* (**e**), *ZmSerPIN* (**f**), and *ZmRIP2* (**g**); concentration of soluble protein (**h**) in the leaves of wild type (WT) B73 plants growing in BX+ or BX- soils ("Conditioning") which were left untreated, sterilized or sterilized and complemented with

microbial extracts ("Soil treatment", +SE, *n*=6-10). Cond. or C, conditioning. Treat or T, treatment; n.s., no significant; FW, fresh weight. The results of two-way ANOVAs are shown. Asterisks indicate significant differences between conditioning genotypes within soil treatments (*$P < 0.05$; **$P < 0.01$; ***$P<0.001$, two-way ANOVAs followed by paired comparisons through FDR-corrected LSMeans).

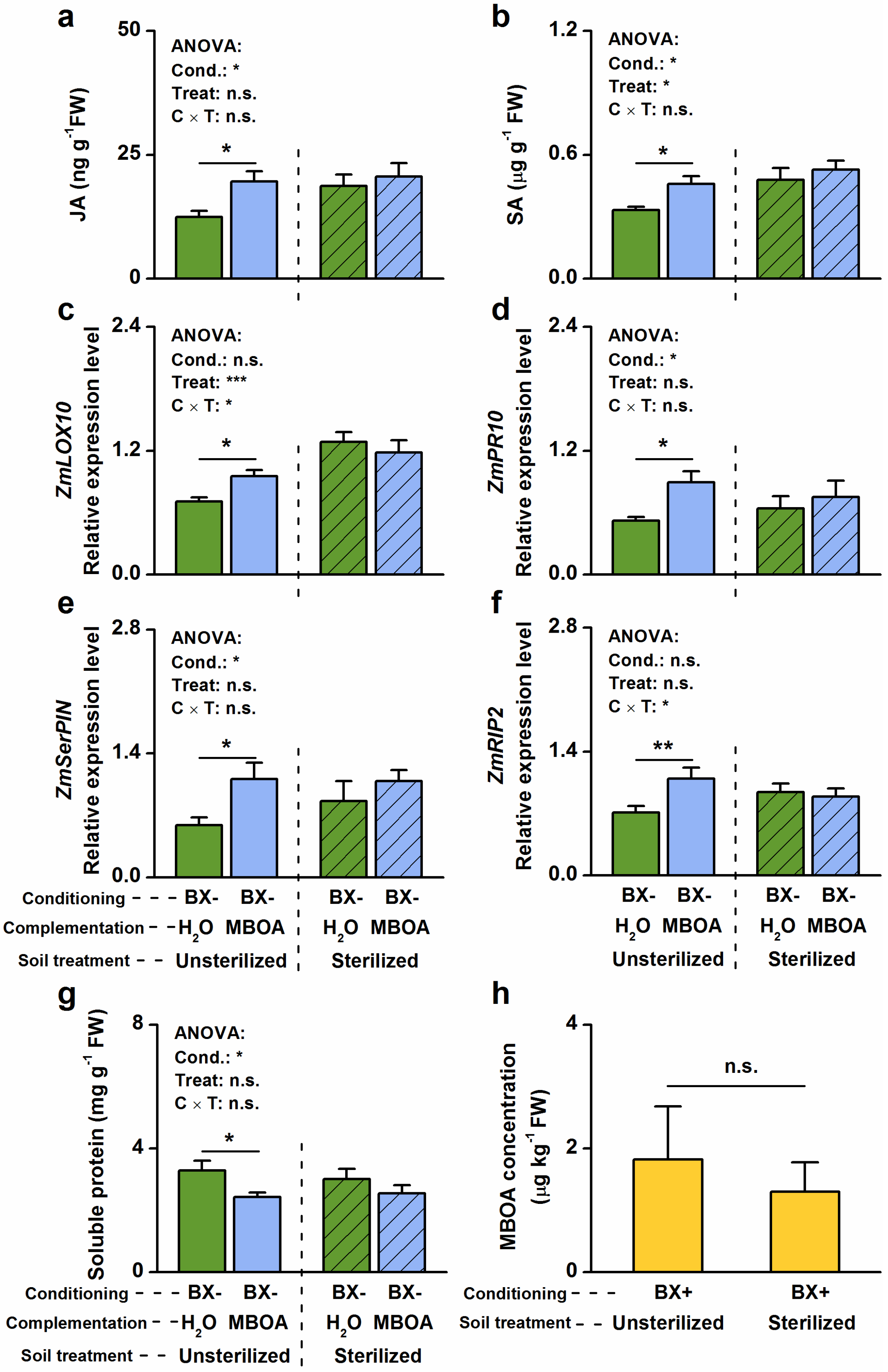

Supplementary Figure 8. Influence of sterilization on 6-methoxy-benzoxazolin-2-one (MBOA)-induced effects on leaf metabolic markers. a-g, Average contents of jasmonic

acid (JA, **a**), and salicylic acid (SA, **b**); expression levels of *ZmLOX10* (**c**), *ZmPR10* (**d**), *ZmSerPIN* (**e**), and *ZmRIP2* (**f**); concentration of soluble protein (**g**) in the leaves of wild type (WT) B73 plants growing in soils previously conditioned by BX-deficient *bx1* mutant plants (BX-) ("Conditioning") which were complemented with water or MBOA ("Complementation") with and without subsequent sterilization ("Soil treatment", +SE, n=11). Cond. or C, conditioning. Treat or T, treatment; n.s., no significant; FW, fresh weight. The results of two-way ANOVAs are shown. Asterisks indicate significant differences between complementation treatments within soil treatments (*P < 0.05; **P < 0.01; ***P<0.001, two-way ANOVAs followed by paired comparisons through FDR-corrected LSMeans). **h**, Average concentration of MBOA in BX+ soil with and without X-ray sterilization (+SE, n=6). No significant difference was found between soil treatments (P > 0.05, Student's *t*-test).

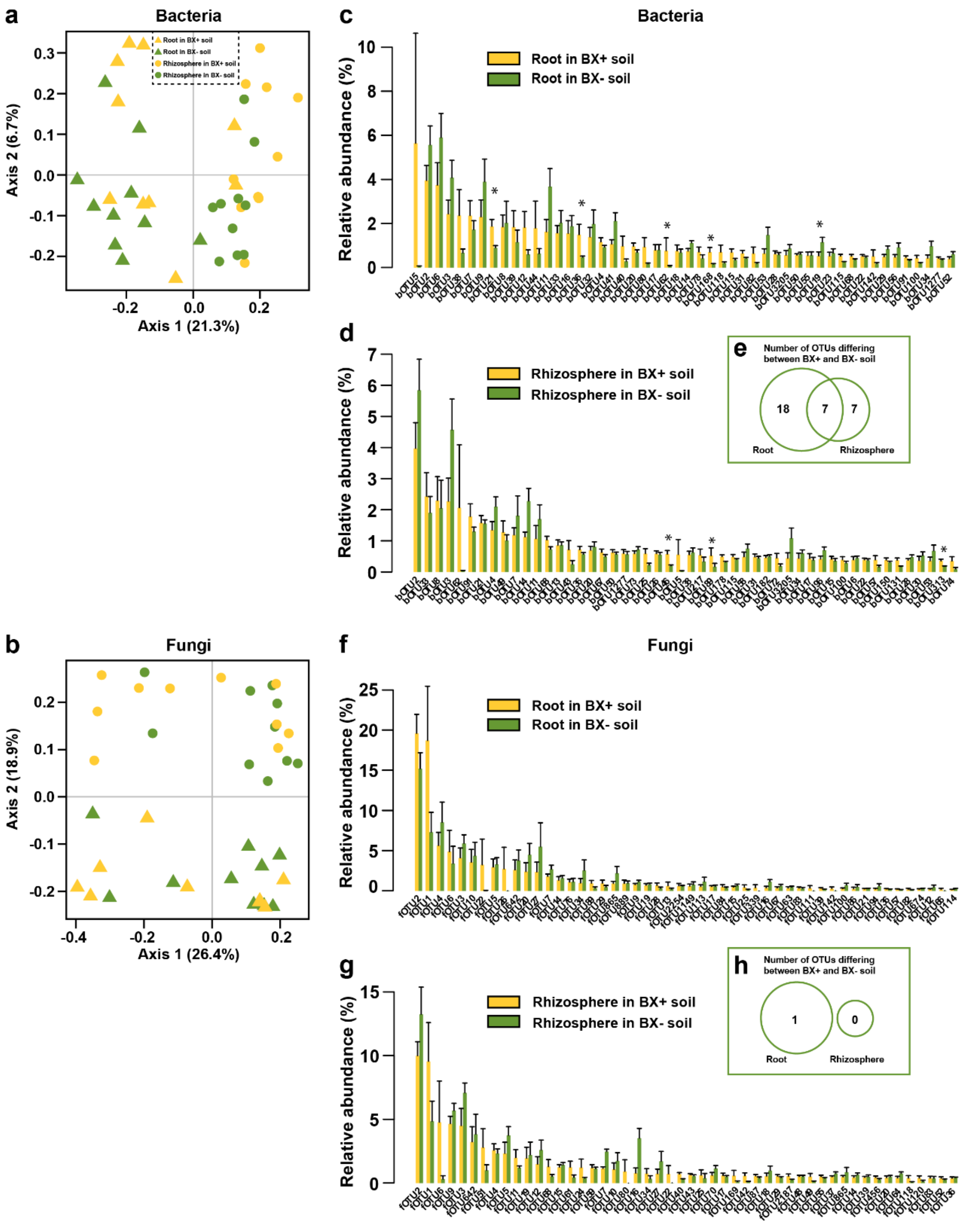

Supplementary Figure 9. Principal Coordinate Analysis of microbiome profiles and individual operational taxonomic units (OTUs) in the root and rhizosphere of the second plant generation. a, Unconstrained ordination of 16S rRNA bacterial gene (**a**) and ITS fungal gene (**b**) profiles based on Bray Curtis distances. Profiles of wild type (WT) B73 rhizosphere and root samples of plants growing in soils previously conditioned by WT (BX+) or BX-deficient *bx1* mutant plants (BX-). Data points represent individual replicate samples (*n*=10). The feedback dataset contained 3,254 and 1,741 bOTUs and fOTUs, of which 184 and 102 had a higher minimal average abundance than 0.1%, respectively. The rank abundance bargraphs (**c, d, f, g**) report the top 50 abundant OTUs. **c, d,** Relative

abundance of bacterial OTUs in the roots (**c**) and rhizosphere (**d**) of wild type (WT) B73 plants grown in BX+ or BX- soils (+SE, n=10). **e,** Number of bacterial OTUs differing between BX+ or BX- soils in root and rhizosphere samples. **f, g,** Relative abundance of fungal OTUs in the roots (**f**) and rhizosphere (**g**) of wild type (WT) B73 plants grown in BX+ or BX- conditioned soils (+SE, n=10). **h,** Number of fungal OTUs differing between BX+ or BX- soils in root and rhizosphere samples. Asterisks indicate significant differences between soil types ($P < 0.05$ (FDR); edgeR's likelihood ratio test).

Supplementary Table 1. Permutational multivariate analysis of variance (PERMANOVA) of microbial profiles from the field experiment.

	Df	SumsOfSqs	MeanSqs	§F.Model	R^2	Pr(>F)	‡Sig
**Bacteria**							
sample_type	2	3.2412	1.62061	17.8290	0.45543	**1e-05**	***
sample_type:genotype	2	0.3306	0.16531	1.8187	0.04646	**0.049**	*
Residuals	39	3.5450	0.09090		0.49811		
Total	43	7.1168			1.00000		
**Fungi**							
sample_type	2	2.6029	1.30146	7.7969	0.27098	**1e-05**	***
sample_type:genotype	2	0.4928	0.24640	1.4761	0.05130	0.071	.
Residuals	39	6.5098	0.16692		0.67772		
Total	43	9.6055			1.00000		

§adonis(formula=bray_curtis.dis ~ sample_type/genotype, data=field, perm.=99999)
‡Significance codes: 0 (***) 0.001 (**) 0.01 (*) 0.05 (.) 0.1

Supplementary Table 2. Pairwise PERMANOVA (upper values) and pairwise BETADISP (observed, lower values) statistic analyses from the field experiment.

PERMANOVA[§‡]<br>BETADISP[§‡]	Bulk soil	WT rhizosphere	WT root	*bx1* rhizosphere
**Bacteria**				
WT rhizosphere	**6.0e-05**<br>**0.010738**	-	-	-
WT root	**6.0e-05**<br>0.346879	**6.0e-05**<br>0.230737	-	-
bx1 rhizosphere	**8.3e-05**<br>**0.012600**	**0.00893**<br>0.816234	**6.0e-05**<br>0.229469	-
bx1 root	**8.6e-05**<br>0.159739	**6.0e-05**<br>0.183835	**0.04377**<br>0.837585	**0.00094**<br>0.119711
**Fungi**				
WT rhizosphere	**0.00015**<br>0.242288	-	-	-
WT root	**0.0001**<br>0.800121	**0.04761**<br>0.261668	-	-
bx1 rhizosphere	**0.00016**<br>**0.037604**	0.251<br>0.744383	**0.002057**<br>0.129127	-
bx1 root	**0.00016**<br>0.135334	**0.00016**<br>0.624146	0.07068<br>0.17080	**0.00165**<br>0.799197

[§] tested between sample groups, permutations=99999
[‡] *P*-values < 0.05 are marked in bold.

Supplementary Table 3. Analysis of soil nutrients, composition and pH in field soils previously conditioned by wild type B73 (BX+) or BX-deficient *bx1* mutant plants (BX-). Mean ± SE; DW, dry weight. No statistical differences were found for any of the measured parameters (Student's *t*-tests, $P > 0.05$)

	field BX+ soil	field BX- soil	unit	replicate
total C	1.3 ± 0.09	1.3 ± 0.04	% mass	*n*=10
total N	0.15 ± 0.008	0.15 ± 0.005	% mass	*n*=10
Ca	2302.9 ± 52.21	2402.2 ± 52.22	mg kg^{-1} DW	*n*=10
K	252.5 ± 9.86	251.4 ± 9.86	mg kg^{-1} DW	*n*=10
Fe	206.7 ± 9.02	209.1 ± 9.02	mg kg^{-1} DW	*n*=10
Mg	104.4 ± 2.88	105.0 ± 2.88	mg kg^{-1} DW	*n*=10
P	55.8 ± 3.26	60.7 ± 3.26	mg kg^{-1} DW	*n*=10
S	14.2 ± 0.28	15.0 ± 0.28	mg kg^{-1} DW	*n*=10
Na	11.7 ± 0.54	11.7 ± 0.54	mg kg^{-1} DW	*n*=10
Cu	10.4 ± 0.13	10.5 ± 0.13	mg kg^{-1} DW	*n*=10
Pb	7.2 ± 0.08	7.1 ± 0.08	mg kg^{-1} DW	*n*=10
Zn	5.4 ± 0.41	4.6 ± 0.41	mg kg^{-1} DW	*n*=10
Ni	3.5 ± 0.04	3.5 ± 0.04	mg kg^{-1} DW	*n*=10
humus	1.9 ± 0.07	1.9 ± 0.038	% mass	*n*=4
sand	28.8 ± 2.04	32.1 ± 2.14	% mass	*n*=4
silt	37.9 ± 1.44	36.2 ± 0.34	% mass	*n*=4
clay	31.3 ± 1.38	29.8 ± 2.11	% mass	*n*=4
corg	1.1 ± 0.04	1.1 ± 0.02	% mass	*n*=4
pH	7.78 ± 0.19	7.78 ± 0.19	pH	*n*=4

Supplementary Table 4. Analysis of soil nutrients, composition and pH in soils conditioned by wild type B73 (BX+) or BX-deficient *bx1* mutant plants (BX-) from the responding plants. Mean ± SE; DW, dry weight. No statistical differences were found for any of the measured parameters (Student's *t*-tests, $P > 0.05$)

	BX+ soil	BX- soil	unit	replicate
total N	0.051 ± 0.0095	0.053 ± 0.0046	% mass	*n*=4
Ca	26582.5 ± 10671.91	16266.8 ± 3543.98	mg kg^{-1} DW	*n*=4
K	41.6 ± 6.45	50.2 ± 3.78	mg kg^{-1} DW	*n*=4
Mg	195.4 ± 44.96	148.0 ± 14.24	mg kg^{-1} DW	*n*=4
P	29.7 ± 1.37	30.9 ± 2.02	mg kg^{-1} DW	*n*=4
humus	0.6 ± 0.07	0.7 ± 0.06	% mass	*n*=4
sand	79.6 ± 21.0	77.8 ± 11.0	% mass	*n*=4
silt	11.7 ± 9.5	11.3 ± 3.5	% mass	*n*=4
clay	8.2 ± 1.1	10.2 ± 0.9	% mass	*n*=4
corg	0.3 ± 0.04	0.4 ± 0.04	% mass	*n*=4
pH	8.08 ± 0.10	8.08 ± 0.09	pH	*n*=4

Supplementary Table 5. Permutational multivariate analyses of leaf markers of wild type (WT) B73 plants growing in soils previously conditioned by WT (BX+) or BX-deficient *bx1* mutant plants (BX-) with or without sterilization and complementation with microbial extracts.

	Df	Variance	F	Pr(>F)	‡Sig
Treatment	2	0.7978	3.8556	**0.001**	***
Conditioning	1	0.7549	7.2967	**0.001**	***
Treatment × Conditioning	2	0.7930	3.8324	**0.001**	***
Residual	45	4.6556			

§Model: formula=tab. scaled ~ conditioning*treatment, data=tab, perm.=999)
‡Significance codes: 0 (***) 0.001 (**) 0.01 (*) 0.05

Supplementary Table 6. *P* values calculated by pairwise comparisons from 999 permutations under the full model for each data set of Supplementary Table 5.

	Unsterilized BX- soil	Unsterilized BX+ soil	Sterilized BX- soil	Sterilized BX+ soil	Sterilized BX- soil & BX- microbial extracts
Unsterilized BX+ soil	**0.0017**				
Sterilized BX- soil	**0.0017**	**0.0017**			
Sterilized BX+ soil	**0.0017**	**0.0017**	0.6840		
Sterilized BX- soil & BX- microbial extracts	**0.0277**	**0.0017**	**0.0017**	**0.0030**	
Sterilized BX+ soil & BX+ microbial extracts	**0.0017**	0.0996	**0.0109**	**0.0112**	**0.0017**

Model: formula=pairwise.factorfit (RDA, tab$conditioning*treatment, xax=1, yax=2, perm.=999)

Supplementary Table 7. Permutational multivariate analysis of variance (PERMANOVA) of microbial profiles from the feedback experiment.

	Df	SumsOfSqs	MeanSqs	§F.Model	R^2	Pr(>F)	‡Sig.
**Bacteria**							
sample_type	1	1.1344	1.13443	8.3443	0.17189	**1e-05**	***
soil	1	0.4379	0.43793	3.2212	0.06636	**7e-04**	***
sample_type:soil	1	0.1332	0.13316	0.9795	0.02018	0.4306	
Residuals	36	4.8943	0.13595		0.74158		
Total	39	6.5998			1.00000		
**Fungi**							
sample_type	1	1.1934	1.19340	7.9745	0.17404	**1e-05**	***
soil	1	0.3098	0.30978	2.0700	0.04518	**0.0362**	***
sample_type:soil	1	0.1158	0.11582	0.7739	0.01689	0.6338	
Residuals	35	5.2379	0.14965		0.76389		
Total	38	6.8569			1.00000		

§adonis(formula=bray_curtis.dis ~ sample_type * soil, data=feedback, perm.=99999)
‡Significance codes: 0 (***) 0.001 (**) 0.01 (*) 0.05

Supplementary Table 8. Pairwise PERMANOVA (upper values) and pairwise BETADISP (observed, lower values) statistic analyses from the feedback experiment.

PERMANOVA§‡<br>BETADISP§‡	BX+ rhizosphere	BX+ root	BX- rhizosphere
**Bacteria**			
BX+ root	**6e-05**<br>0.371450	-	-
BX- rhizosphere	**0.021**<br>0.066819	**7.5e-05**<br>**0.035516**	-
BX- root	**6e-05**<br>0.143615	**0.020**<br>0.061845	**6e-05**<br>0.798337
**Fungi**			
BX+ root	**0.001695**<br>0.931650	-	-
BX- rhizosphere	0.08596<br>**0.023678**	**6e-05**<br>0.054050	-
BX- root	**0.00014**<br>0.307177	0.3212<br>0.362910	**0.00014**<br>0.275500

§ tested between sample groups, permutations=99999
‡ *P*-values < 0.05 are marked in bold.
