# Supplementary material for: Root exudate metabolites drive plant-soil feedbacks on growth and defense by shaping the rhizosphere microbiota
Source: Nat Commun. 2018 Jul 16;9:2738. doi: 10.1038/s41467-018-05122-7 (PMC6048113; doi:10.1038/s41467-018-05122-7)
Supplement: Supplementary file 3 — Description of Additional Supplementary Files [file 41467_2018_5122_MOESM3_ESM.pdf]

Description of Additional Supplementary Files:

Supplementary Movie 1. Root sampling. Employing scissors to sample the ~10 cm fragment corresponding to the soil depth between −5 and −15 cm.

Supplementary Data 1. Experimental Design. Mapping of samples and utilized barcodes.

Supplementary Data 2. Bioinformatics script. The archive contains the command line script, including all individual parameters and support files.

Supplementary Data 3. Microbiome analysis in R. The archive comprises a PDF report (the R-markdown output) that summarizes the microbiome analysis in R. This report also provides additional background information, a description of the sequencing effort as well as explanations and justifications for the analysis logic.

Supplementary Data 4. Microbiome statistics. This file lists for each sample type the taxonomies and statistic details (fold change (as log), abundance (as counts per million, log), edgeR's likelihood ratio statistic with corresponding FDR corrected *P*-values) of the differentially abundant OTUs (field experiment: B73 vs. *bx1* plants; feedback experiment: B73-conditioned [BX+] vs. *bx1*-conditioned [BX-] soils).
