# Supplementary material for: Root exudate metabolites drive plant-soil feedbacks on growth and defense by shaping the rhizosphere microbiota
Source: Nat Commun. 2018 Jul 16;9:2738. doi: 10.1038/s41467-018-05122-7 (PMC6048113; doi:10.1038/s41467-018-05122-7)
Supplement: Supplementary file 7 — Supplementary Data 3 [file 41467_2018_5122_MOESM7_ESM.zip › Supplementary Data 3.zip!/Supplementary Data 3/Supplementary Data 3/Hu_et_al_bacteria_community_analysis_in_R.pdf]

# Hu et al. (2018), bacteria community analysis in R

*Klaus Schlaeppi*

*2018-04-18*

## Contents

**Background** **2**

**Overview all data** **2**
Sequencing effort . . . 2
Community similarities between field and feedback experiments . . . 3

**Analysis field experiment** **4**
Alpha diversity . . . 4
Beta diverstiy . . . 5
PCoA - unconstrained ordination . . . 5
CAP - constrained ordination . . . 5
PERMANOVA . . . 6
Identification of differentially abundant OTUs . . . 8

**Analysis feedback experiment** **11**
Alpha diversity . . . 11
Beta diverstiy . . . 12
PCoA - unconstrained ordination . . . 12
CAP - constrained ordination . . . 12
PERMANOVA . . . 15
Identification of differentially abundant bOTUs . . . 16

# Background

We have sequenced the bacteria communities of a field experiment and a feedback experiment. We have prepared two separate libraries, sequenced them in separate sequencing runs and the raw data is stored in two separate ENA archives. The raw data of both sequencing runs were processed separetely using the same bioinformatic pipeline (Database S2). The high quality sequences from both experiments were combined for inferring bacterial OTUs (bOTUs) and generation of a common bOTU table (Database S2). The analysis of the bacteria communities in R requires the following input files:
- Experimental design: *Database_S1.txt* (save Database S1 as tab-delimited text file)
- OTU table: *both_exps_trimmed_qfiltered_renamed_l360_sort_derep_ab5_otu_chimerafree.tab*
- Taxonomy: *SILVA_tax_forR.txt*

# Overview all data

## Sequencing effort

For the field experiment we generated a total of 1'377'716 high quality sequences with a median sequence number of 24'195 sequences per sample (range: 6'062 to 132'089). The feedback experiment yielded a total of 4'236'141 high quality sequences with a median sequence number of 86'438 sequences per sample (range: 4'782 to 349'584).

The field and feedback experiments comprise the following sample groups:

***Field:*** 5 groups with soil samples; rhizosphere and root samples of both wildtype B73 or *bx1* mutant plants.

***Feedback:*** 4 groups with rhizosphere and root samples of wildtype B73 plants that were grown both on B73 (BX+) or *bx1* (BX-) conditioned soils.

The mean sequence numbers per sample group are shown in the plot below.

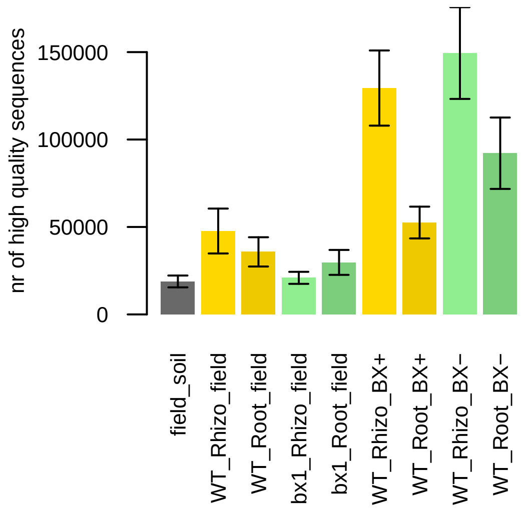

To decide on how to normalize the data we followed the recommendation of Weiss et al. (2015, PeerJ) and we inspected whether there are differences in sequencing depths between the different sample groups utilizing the non-parametric Kruskal-Wallis Test.

```
##
##  Asymptotic Kruskal-Wallis Test
##
## data:  colSums(bDAT) by
##   design$groups (field_soil, WT_Rhizo_field, WT_Root_field, bx1_Rhizo_field, bx1_Root_field, WT_Rhiz[illegible]
## chi-squared = 51.883, df = 8, p-value = 1.773e-08
```

Since we found significant differences in sequence numbers between the sample groups we decided to employ rarefication for normalization of the data. Rarefication disrupts most reliably possible effects between sample groups that are due to sampling depth (see Weiss et al., 2015 PeerJ).

## Community similarities between field and feedback experiments

Please note: the setups of field and feedback experiments differ substantially. The field experiment used ***full field soil*** and ***natural conditions*** while the feedback experiment utilized ***sand-amended field soil in pots*** and ***controlled greenhouse conditions***. These experimental factors are likely to confound such a comparison. Only for curiosity, we inspect here the community similarities between both experiments using the r-package 'phyloseq' and as input, we employed rarefied data (rarefied to the minimal sampling depth of both experiment, 4'782 sequences per sample). The plot below consists of an Principal Coordiante Analysis (PCoA) with Bray-Curtis distances utilizing the data of both the field and the feedback experiments and shows that the samples differ between the two experiments. Hence, because of confounding experimental factors we renounced comparing the field with the feedback experiment for the analysis that we present in the manuscript.

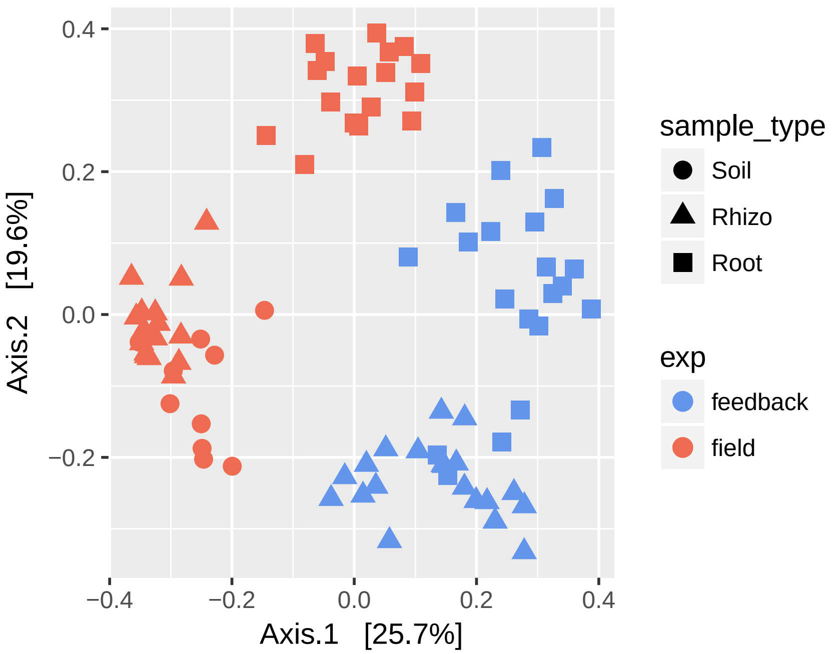

# Analysis field experiment

The field experiment consistet of a total of 44 samples representing 5 sample groups (soil samples and rhizosphere and root samples of both wildtype B73 or *bx1* mutant plants). The number of replicates per sample group is given below.

| field_soil | WT_Rhizo | WT_Root | bx1_Rhizo | bx1_Root |
|---|---|---|---|---|
| 10 | 10 | 10 | 7 | 7 |

## Alpha diversity

We examined alpha (within sample) diversity patterns of the field experiment data using the indices of Chao's estimate of richness, Shannon diversity and Pielou's evenness. We were interested in alpha diversity patterns between sample types and if BX exudation would affect microbial diversity. We utilized the 'estimate_richness' function of the r-package 'phyloseq' and employed rarefied data (rarefied to the minimal sampling depth in the field experiment, 6'062 sequences per sample) as input. The plots below report Chao-1 richness, Shannon diversity and Pielou's evenness.

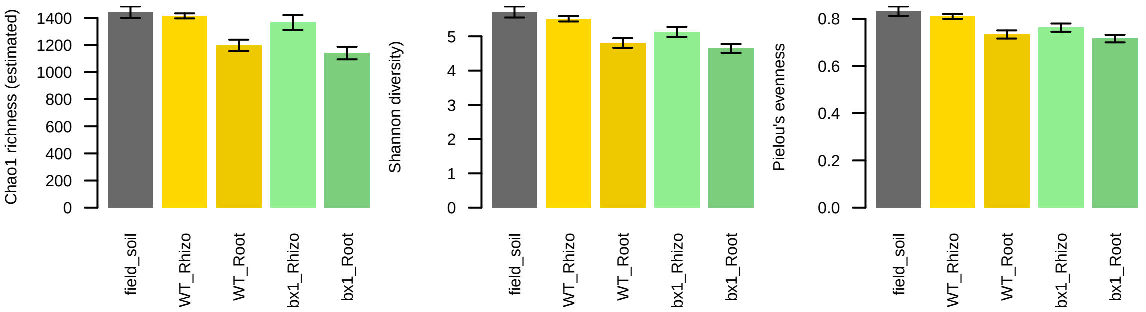


Statistic examination using Analysis of Variance (ANOVA, model with ~ *sample type* / *plant genotype*) yielded significant differences between *sample type* (soil, rhizosphere and root) for Chao-1 richness $P$=2.16e-10, Shannon diversity $P$=7.82e-08 and Pielou's evenness $P$=9.93e-06. Althought, all three mean diversity indices tend to be slightly lower in *bx1*(B73) compared to B73 (both in roots and rhizosphere samples), the F-tests for the factor *plant genotype* (B73 or *bx1*(B73)) were *not* significant (Chao-1 richness $P$=1.38e-01, Shannon diversity $P$=5.24e-02 and Pielou's evenness $P$=1.32e-01). Therefore, no further examination with a post-hoc test was conducted.

## Beta diverstiy

### PCoA - unconstrained ordination

In a first step, we examined the field experiment data for beta diversity patterns. In particular, we were interested in community similarites between the sample groups. We utilized the r-package 'phyloseq' and employed rarefied data (rarefied to the minimal sampling depth in the field experiment, 6'062 sequences per sample) as input. The plot below reports the PCoA (unconstrained ordination) based on Bray Curtis distances (Supplementary Figure 1).

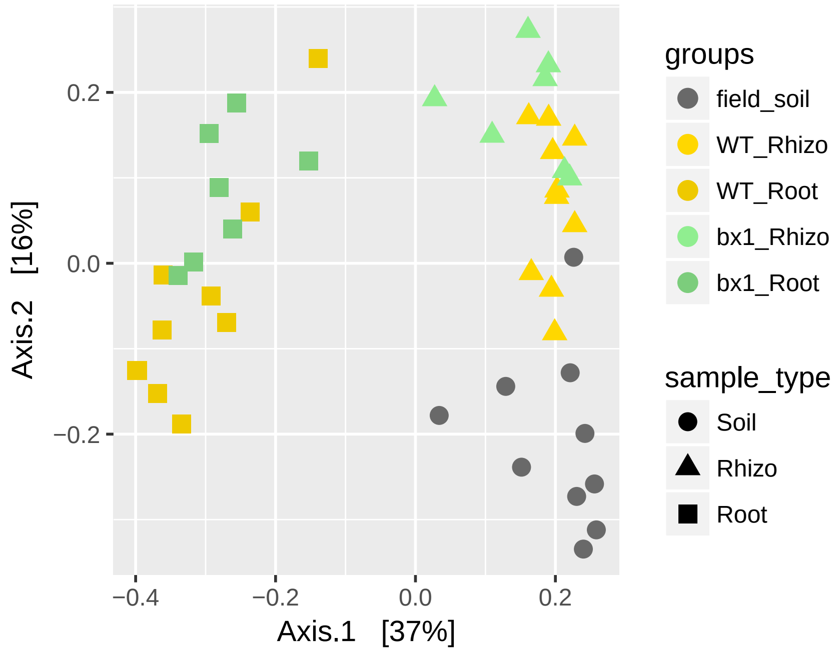


### CAP - constrained ordination

We then used constrained ordination (partial canonical analysis of principal coordinates; CAP) to evaluate how the variation in the dataset can be partitioned to the experimental factors *sample type* (soil, rhizosphere and root) and *plant genotype* (B73 or *bx1*(B73)). A model with nesting *plant genotype* within the factor *sample type* was used for the CAP (~*sample type/plant genotype*). We validated the significance of the model with ANOVA using 99'999 permutations (table below). The plot below reports the CAP based on Bray Curtis distances (Figure 1 in the manuscript) with the title stating the constrained factor(s), their amount of explained variation and their statistic significance.

| | Df | SumOfSqs | F | Pr(>F) |
|---|---|---|---|---|
| **Model** | 4 | 3.572 | 9.824 | 1e-05 |
| **Residual** | 39 | 3.545 | NA | NA |

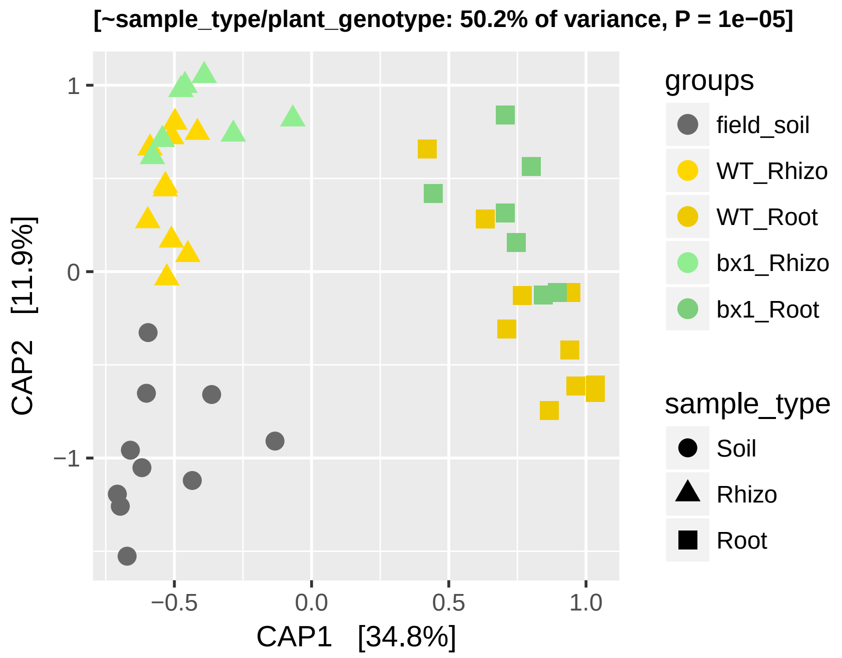


Conclusions: Both PCoA and CAP reveal that the root microbiom is more dissimilar than the rhizoshpere and soil microbiomes. In addition, the exudation of BXs has a effect on rhizosphere and root communities, both with same direction.

### PERMANOVA

We utilized Permutational Multivariate Analysis of Variance (PERMANOVA) to partition and quantify the effects of the experimental factors *sample type* (soil, rhizosphere and root) and *plant genotype* (B73 or *bx1* (B73)). For this we utilized the function 'adonis' of the r-package 'vegan' with a Bray Curtis distance matrix as input and running 99'999 permutations. Again, we fitted a model with nesting *plant genotype* within the factor *sample type* (*~sample type/plant genotype*). The PERMANOVA output below is reported as Supplementary Table 1.

Table 3: Table continues below

| | Df | SumsOfSqs | MeanSqs | F.Model | R2 | Pr(>F) |
|---|---|---|---|---|---|---|
| **sample_type** | 2 | 3.241 | 1.621 | 17.83 | 0.4554 | 1e-05 |
| **sample_type:plant_genotype** | 2 | 0.3306 | 0.1653 | 1.819 | 0.04646 | 0.04879 |
| **Residuals** | 39 | 3.545 | 0.0909 | NA | 0.4981 | NA |
| **Total** | 43 | 7.117 | NA | NA | 1 | NA |

We also conducted pairwise comparison between the five sample groups (soil, WT rhizosphere, bx1 rhizoshpere, WT root and bx1 root samples) utilizing the function 'pairwise.perm.manova' of the r-package 'RVAideMemoire'

with the same Bray Curtis distance matrix as input and running 99'999 permutations. We fitted a simple model with the five sample groups as factor. The pairwise PERMANOVA output below is reported in Supplementary Table 2. The reported *P*-values are adjusted for false discovery rate (fdr).

| | field_soil | WT_Rhizo | WT_Root | bx1_Rhizo |
|---|---|---|---|---|
| **WT_Rhizo** | 6e-05 | NA | NA | NA |
| **WT_Root** | 6e-05 | 6e-05 | NA | NA |
| **bx1_Rhizo** | 8.333e-05 | 0.008933 | 6e-05 | NA |
| **bx1_Root** | 8.571e-05 | 6e-05 | 0.04377 | 0.0009375 |

Differences in beta-diversity can be driven by true biological differences between groups, or differences in group dispersion (variance), or both (Anderson MJ. A new method for non parametric multivariate analysis of variance. Austral Ecol. 2001;26:32–46.). Therefore, we tested for differences in dispersion between the five sample groups (soil, WT rhizosphere, bx1 rhizoshpere, WT root and bx1 root samples) using the functions 'betadisper' and 'permutest' of the r-package 'vegan'. The pairwise BETADISP output below is reported in Supplementary Table 2. Lack of significance in the dispersion tests supports that differences between the sample groups are driven by true biological differences and do not present an artifact due to differences in dispersion.

| | observed | permuted |
|---|---|---|
| **field_soil-WT_Rhizo** | 0.01074 | 0.00762 |
| **field_soil-WT_Root** | 0.3469 | 0.358 |
| **field_soil-bx1_Rhizo** | 0.0126 | 0.00983 |
| **field_soil-bx1_Root** | 0.1597 | 0.1616 |
| **WT_Rhizo-WT_Root** | 0.2307 | 0.235 |
| **WT_Rhizo-bx1_Rhizo** | 0.8162 | 0.8241 |
| **WT_Rhizo-bx1_Root** | 0.1838 | 0.185 |
| **WT_Root-bx1_Rhizo** | 0.2295 | 0.2338 |
| **WT_Root-bx1_Root** | 0.8376 | 0.8451 |
| **bx1_Rhizo-bx1_Root** | 0.1197 | 0.1158 |

*In conclusion:* Pairwise PERMANOVA revealed that bacterial communities differ significantly between the five sample groups (soil, WT rhizosphere, bx1 rhizoshpere, WT root and bx1 root samples). The pairwise BETADISP tests supported (significant differences between the sample groups are driven by true biological differences and not due to differences in dispersion) most of these comparisons, including WT vs. bx1 rhizospheres as well as WT vs. bx1 roots. The comparisons between soil and rhizosphere samples presented an exception because the differences arise from differences in dispersion of the data. Hence, soil and rhizosphere compartments should not be considered significantly different from each other.

## Identification of differentially abundant OTUs

In a second step, we identified both in rhizosphere and root samples OTUs that differed in their relative abundance between wildtype B73 and mutant *bx1*(B73) plants. For this analysis we focussed on the abundant OTUs similar to Bai et al. (2016, Nature). We defined abundant OTUs as OTUs that have a minimal average abundance of 0.1% in the dataset. We utilized the r-package 'edgeR' for this analysis as particular strengths of this tool are the ability to estimate biological dispersion between replicate samples, and the performance of exact tests of significance that are suitable for small counts. We estimated dispersion by weighted likelihood empirical Bayes, modeled the OTU abundances as a function for the five sample groups and we contrasted the two genotypes, both for rhizosphere and root samples for the likelyhood ratio tests. P-values were adjusted according to Benjamini and Hochberg (1995).

Of the 3'107 bOTUs in the field dataset, 184 had a higher minimal average abundance than 0.1%.

### *Rhizosphere samples: bOTUs differing between B73 and* bx1 *plants*

Below we list the direction and taxonomies of the differentially abundant bOTUs (between B73 and *bx1* plants) in the rhizosphere compartment. This information is documented in the Database S4.

| lower in WT | unchanged | higher in WT |
|---|---|---|
| 4 | 174 | 6 |

| | phylum | genus |
|---|---|---|
| **bOTU84** | Proteobacteria | Methylotenera |
| **bOTU62** | Proteobacteria | Pantoea |
| **bOTU79** | Bacteroidetes | Flavobacterium |
| **bOTU618** | Proteobacteria | unassigned |
| **bOTU181** | Proteobacteria | Cellvibrio |
| **bOTU69** | Bacteroidetes | Flavobacterium |
| **bOTU314** | Verrucomicrobia | uncultured |
| **bOTU246** | Proteobacteria | uncultured |
| **bOTU173** | Proteobacteria | Steroidobacter |
| **bOTU322** | Acidobacteria | uncultured Acidobacteria bacterium |

The 50 most abundant bOTUs in the rhizosphere compartment are presented in the rank abundance plot below. The BX-dependent OTUs among the Top50 rhizosphere OTUs are marked with an asterisk. This graph was used for the Supplementary Figure 1.

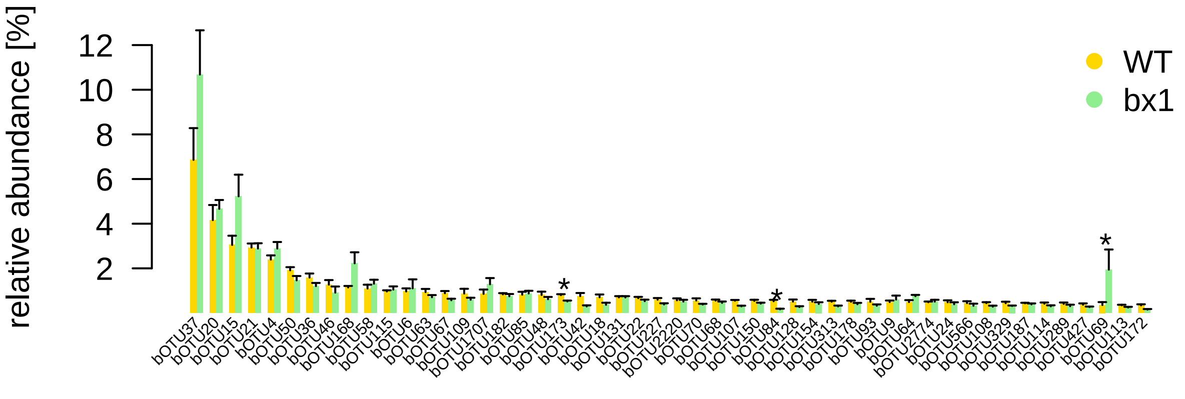

### ***Root samples: bOTUs differing between B73 and* bx1 *plants***

Below we list the direction and taxonomies of the differentially abundant bOTUs (between B73 and *bx1* plants) in the root compartment. This information is comprised in the Database S4.

| lower in WT | unchanged | higher in WT |
|---|---|---|
| 8 | 169 | 7 |

| | phylum | genus |
|---|---|---|
| **bOTU62** | Proteobacteria | Pantoea |
| **bOTU15** | Proteobacteria | Pseudomonas |
| **bOTU79** | Bacteroidetes | Flavobacterium |
| **bOTU889** | Actinobacteria | Actinoplanes |
| **bOTU118** | Proteobacteria | Rhizobium |
| **bOTU1612** | Proteobacteria | Pseudomonas |
| **bOTU233** | Proteobacteria | Delftia |
| **bOTU12** | Proteobacteria | Rhizobium |
| **bOTU315** | Bacteroidetes | Flavobacterium |
| **bOTU202** | Actinobacteria | uncultured |
| **bOTU52** | Actinobacteria | Nocardioides |
| **bOTU353** | Actinobacteria | Kineosporia |
| **bOTU356** | Proteobacteria | Haliangium |
| **bOTU175** | Actinobacteria | unassigned |
| **bOTU133** | Actinobacteria | uncultured microorganism |

The 50 most abundant bOTUs in the root compartment are presented in the rank abundance plot below. The BX-dependent OTUs among the Top50 root bOTUs are marked with an asterisk. This graph was used for the Supplementary Figure 1.

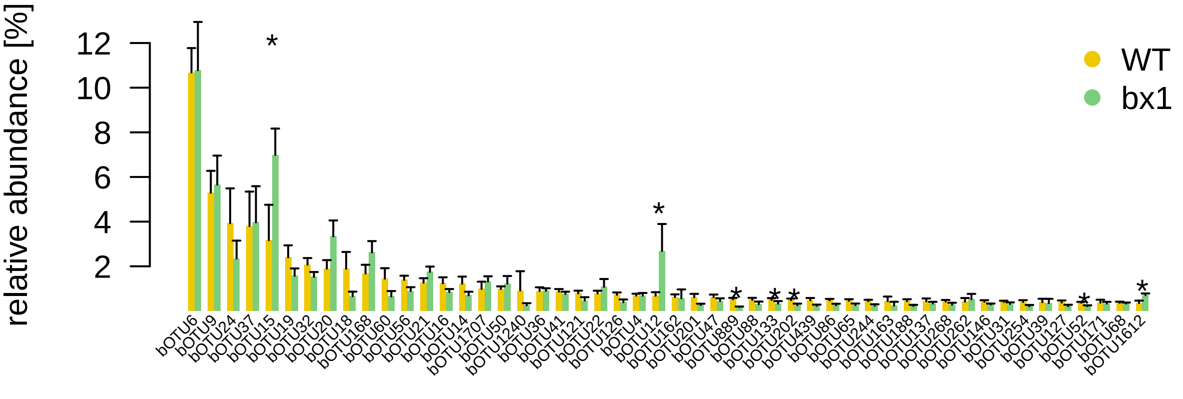

***BX-dependent bOTUs: rhizosphere vs. root compartments***

We then compared the differentially abundant bOTUs between B73 and *bx1* plants of the rhizosphere compartment with the ones from the root samples. We found that most bOTUs were senstive to the exudation of BX in a compartment-specific manner (Venn diagram below, this plot was incorporated into the Supplementary Figure 1).

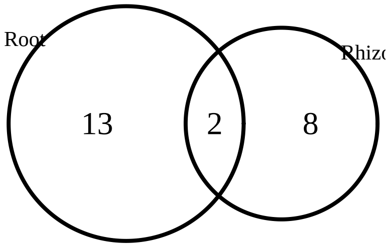


The bargraphs below report the two bOTUs of the intersection - bOTU62 and bOTU79 - that were BX-dependent in both compartments.

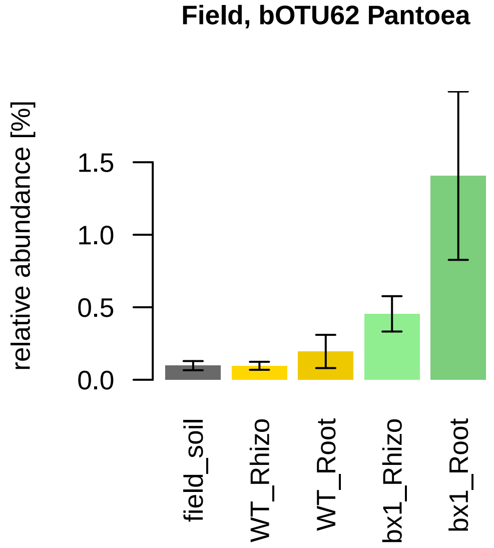


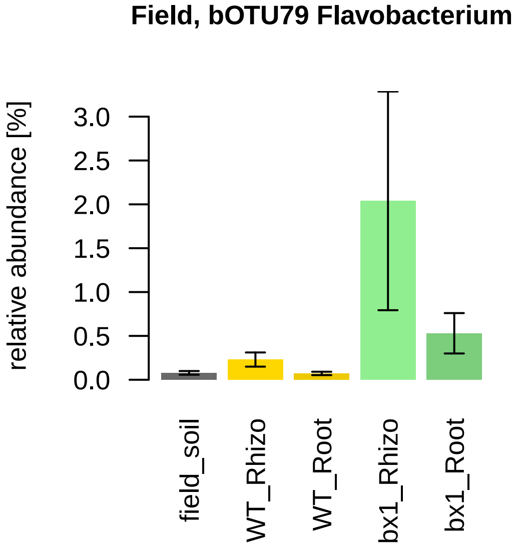

# Analysis feedback experiment

*Note*: we followed the same logic for the analysis of the feedback experiment and we utilized the same r-packages and code as we did for the field experiment.

The feedback experiment consistet of a total of 40 samples representing 4 sample groups (rhizosphere and root samples of wildtype B73 plants that were grown both on B73 (BX+) or *bx1* (BX-) conditioned soils). The number of replicates per sample group is given below.

| Rhizo_WT_BX+ | Root_WT_BX+ | Rhizo_WT_BX- | Root_WT_BX- |
|---|---|---|---|
| 10 | 10 | 10 | 10 |

## Alpha diversity

We examined alpha (within sample) diversity patterns of the feedback experiment data using the indices of Chao's estimate of richness, Shannon diversity and Pielou's evenness. We were interested in alpha diversity patterns between sample types and if BX conditioning would affect microbial diversity. We utilized the 'estimate_richness' function of the r-package 'phyloseq' and employed rarefied data (rarefied to the minimal sampling depth in the feedback experiment, 4'782 sequences per sample) as input. The plots below report Chao-1 richness, Shannon diversity and Pielou's evenness.

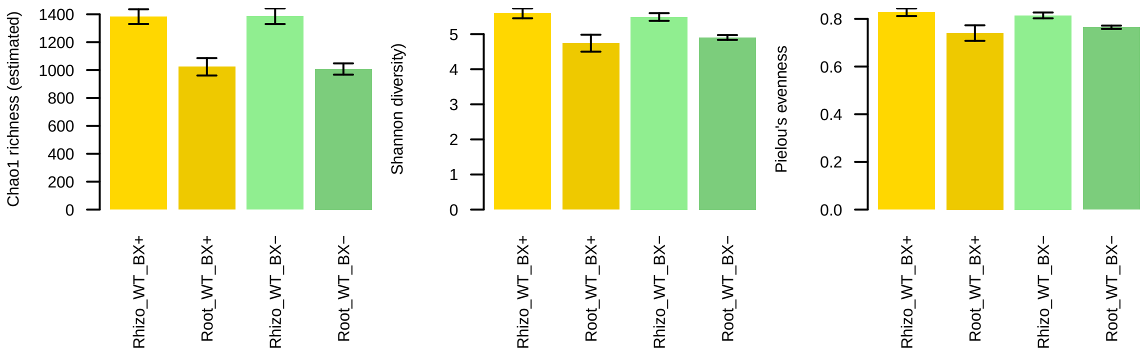


Statistic examination using Analysis of Variance (ANOVA, model with ~ *sample type* x *soil conditioning*) yielded significant differences between *sample type* (rhizosphere and root) for Chao-1 richness $P$=5.18e-09, Shannon diversity $P$=4.29e-07 and Pielou's evenness $P$=1.2e-03 but not *soil conditioning* (BX+ or BX-; from previous plants B73 or *bx1*, respectively): Chao-1 richness $P$=9.58e-01, Shannon diversity $P$=6.05e-01 and Pielou's evenness $P$=7.8e-01. Also the interaction term did not reveal significant differences (Chao-1 richness $P$=6.78e-01, Shannon diversity $P$=8.53e-01 and Pielou's evenness $P$=3.4e-01).

## Beta diverstiy

### PCoA - unconstrained ordination

We first examined the feedback experiment data for beta diversity patterns between the sample groups. The plot below was used for the Supplementary Figure 9 and reports the PCoA (unconstrained ordination) based on Bray Curtis distances (Input: rarefied data at sampling depth 4'782 sequences per sample).

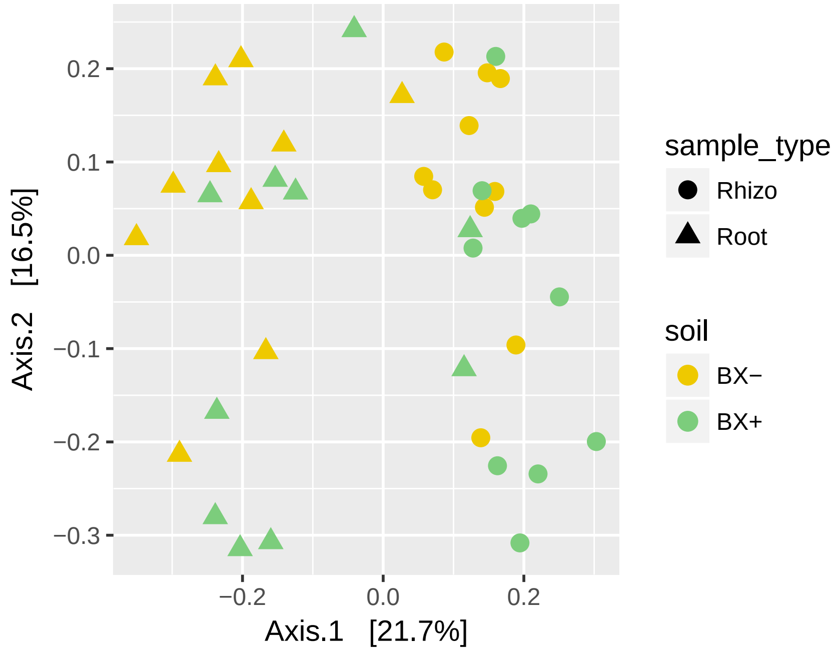


Conclusion: We confirm that root and rhizosphere microbioms are dissimilar (PCo Axis 1). Within each sample type, the type of previous soil conditioning (BX+ or BX-) appears to separate the bacteria communities (PCo Axis 2), both with same direction!.

### CAP - constrained ordination

We then also conducted a CAP analysis to evaluate how the variation in the dataset can be partitioned to the experimental factors *sample type* (rhizosphere and root) and *soil conditioning* (BX+ or BX-). We fitted the same model as with PERMANOVA (~ *sample type* * *soil conditioning*) and validated its significance with ANOVA using 99'999 permutations (table below).

| | Df | SumOfSqs | F | Pr(>F) |
|---|---|---|---|---|
| **Model** | 3 | 1.574 | 4.115 | 1e-05 |
| **Residual** | 36 | 4.59 | NA | NA |

For the feedback experiment, we explored in addition to the sample scores also the bOTU scores of the CAP. The bOTU scores in such a bi-plot analysis present orthogonal projections for each bOTU that fit the sample distribution in the ordination space. In simple terms, the bi-plot analysis reveals bOTUs that contribute to explain the sample distribution pattern in the constrained ordination. We did not perform a bi-plot analysis for the field experiment data, as there the occurrence of soil samples obscures meaningful interpretations with respect to *plant geotype*.

Here, we subsetted the OTU table to bOTUs that belong to the 6 most abundant phyla (this was done as preparation for the subsequent bi-plot analysis; otherwise the bi-plot becomes unclear with bOTUs of too

many phyla). The sample scores plot based on bOTUs of 6 most abundant Phyla differes only marginally compared to a plot with OTUs of all phyla in the dataset. The plot below reports the sample scores of the CAP based on Bray Curtis distances and is presented as Figure 7 in the manuscript. The plot title states the constrained factor(s), their amount of explained variation and their statistic significance.

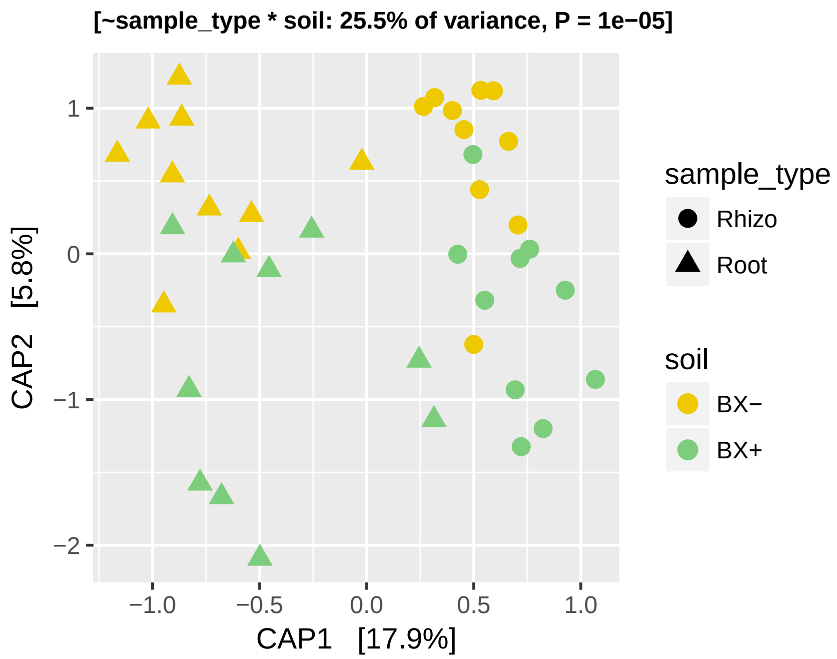


Below is the plot with the OTU scores of the CAP (presented in Figure 7). Please note, the coordiantes of the sample and the OTU plot are congruent. We defined "explanatory" OTUs as bOTUs based on their position along CAP axes 1 ( < -0.1 or > 0.1 ) and CAP axis 2 ( < -0.1 or > 0.1 ). "Explanatory" OTUs are indicated in the bi-plot with their OTU ID.

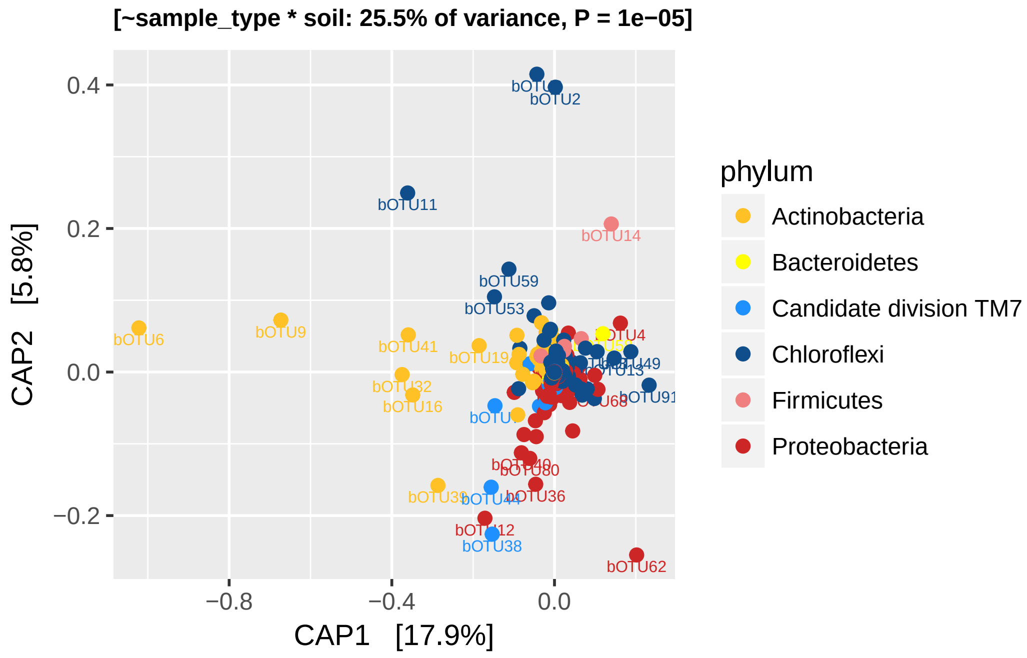

Conclusions: Actinobacteria largely discriminate bacterial communities of rhizosphere and root compartments. In both compartments, subsets of Proteobacteria, Chloroflexi and TM7 differ between BX+ and BX-conditioned soils.

The bargraphs below present a few “explanatory” bOTUs of the CAP and they illustrate how to read bi-plot graphs. One example for each cardinal point of the ordination space was chosen.

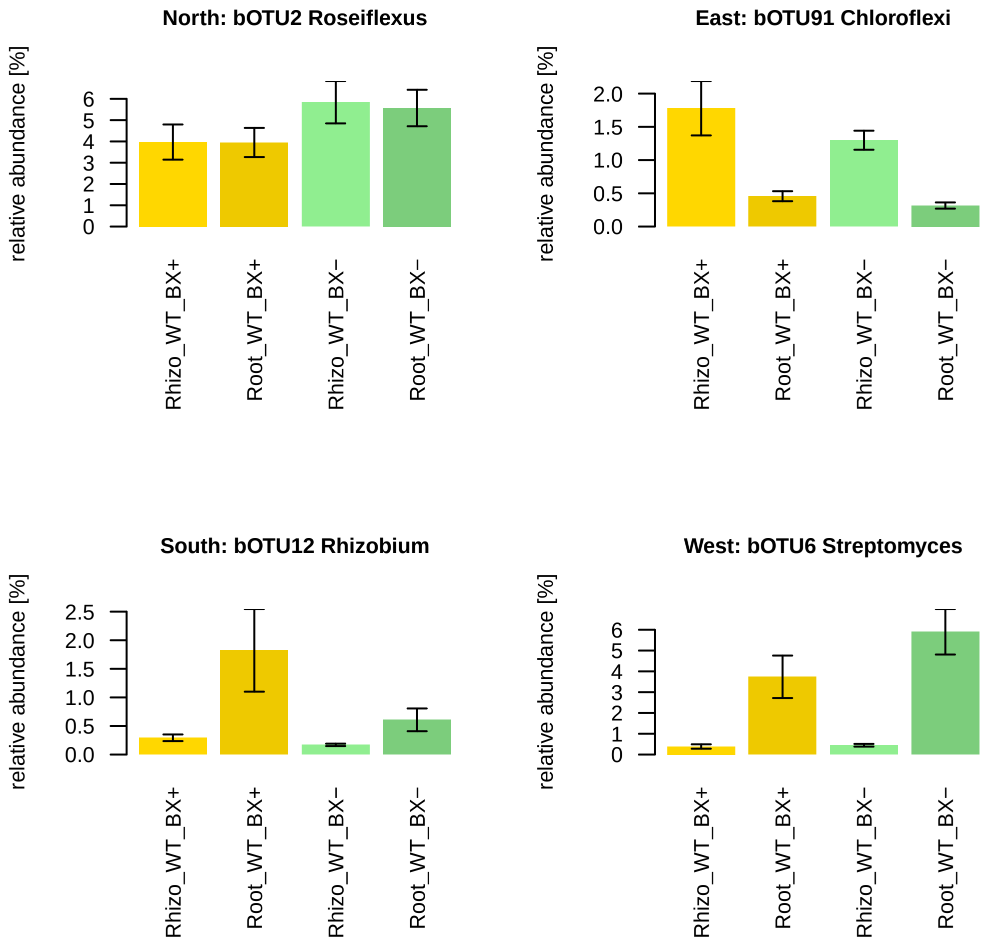

**PERMANOVA**

PERMANOVA was used to partition and quantify the effects of the experimental factors *sample type* (rhizosphere and root) and *soil conditioning* (BX+ or BX-; from previous plants B73 or *bx1*, respectively). A Bray Curtis distance matrix was used as input, we runned 99'999 permutations and fitted a model with interaction between the factors *sample type* and *soil conditioning* (~ *sample type* * *soil conditioning*). The PERMANOVA output below is reported in Supplementary Table 7.

| | Df | SumsOfSqs | MeanSqs | F.Model | R2 | Pr(>F) |
|---|---|---|---|---|---|---|
| **sample__type** | 1 | 1.134 | 1.134 | 8.344 | 0.1719 | 1e-05 |
| **soil** | 1 | 0.4379 | 0.4379 | 3.221 | 0.06636 | 7e-04 |
| **sample__type:soil** | 1 | 0.1332 | 0.1332 | 0.9795 | 0.02018 | 0.4306 |
| **Residuals** | 36 | 4.894 | 0.136 | NA | 0.7416 | NA |
| **Total** | 39 | 6.6 | NA | NA | 1 | NA |

We also conducted pairwise comparison between the sample groups (rhizosphere and root, with and without BX conditioning) utilizing the function 'pairwise.perm.manova' of the r-package 'RVAideMemoire' with the same Bray Curtis distance matrix as input and running 99'999 permutations. We fitted a simple model with the four sample groups as factor. The pairwise PERMANOVA output below is reported in Supplementary Table 8. The reported *P*-values are adjusted for false discovery rate (fdr).

| | Rhizo__WT__BX+ | Root__WT__BX+ | Rhizo__WT__BX- |
|---|---|---|---|
| **Root__WT__BX+** | 6e-05 | NA | NA |
| **Rhizo__WT__BX-** | 0.02076 | 7.5e-05 | NA |
| **Root__WT__BX-** | 6e-05 | 0.01889 | 6e-05 |

Differences in beta-diversity can be driven by true biological differences between groups, or differences in group dispersion (variance), or both (Anderson MJ. A new method for non parametric multivariate analysis of variance. Austral Ecol. 2001;26:32–46.). Therefore, we tested for differences in dispersion between the sample groups using the functions 'betadisper' and 'permutest' of the r-package 'vegan'. The pairwise BETADISP output below is reported in Supplementary Table 8. Lack of significance in the dispersion tests supports that differences between the sample groups are driven by true biological differences and do not present an artifact due to differences in dispersion.

| | observed | permuted |
|---|---|---|
| **Rhizo__WT__BX+-Root__WT__BX+** | 0.3714 | 0.3871 |
| **Rhizo__WT__BX+-Rhizo__WT__BX-** | 0.06682 | 0.05989 |
| **Rhizo__WT__BX+-Root__WT__BX-** | 0.1436 | 0.1441 |
| **Root__WT__BX+-Rhizo__WT__BX-** | 0.03552 | 0.02908 |
| **Root__WT__BX+-Root__WT__BX-** | 0.06185 | 0.05611 |
| **Rhizo__WT__BX–Root__WT__BX-** | 0.7983 | 0.809 |

*In conclusion:* PERMANOVA identified significant effects by the experimental factors *sample type* (rhizosphere and root) and *soil conditioning* (BX+ or BX-), which is confirmed by the pairwise tests revealing that all bacterial communities are significantly different between the sample groups. The pairwise BETADISP tests supported most of these comparisons except the comparisons between BX+ root and BX- rhizosphere samples where the differences arise from differences in dispersion of the data and therefore should not be considered significantly different from each other.

## Identification of differentially abundant bOTUs

In a second step, we identified both in rhizosphere and root samples bOTUs that differed in their relative abundance between the conditioned soils *BX+* (B73 as previous plant genotype) and *BX-* (mutant *bx1* as previous plant). Analogous to the above, we focussed on the abundant bOTUs, we utilized 'edgeR', we modeled the bOTU abundances as a function for the four sample groups and we inspected the contrasts between the two genotypes both in rhizosphere and root samples. P-values were adjusted according to Benjamini and Hochberg (1995).

Of the 3'254 bOTUs in the feedback dataset, 184 had a higher minimal average abundance than 0.1%.

### ***Rhizosphere samples: bOTUs differing between BX+ and BX- soils***

Below we list the direction and taxonomies of the differentially abundant bOTUs (between BX+ and BX- soils) in the rhizosphere compartment. This information is documented in the Database S4.

| higher in BX+ | unchanged | higher in BX- |
|---|---|---|
| 6 | 170 | 8 |

| | phylum | genus |
|---|---|---|
| **bOTU59** | Chloroflexi | Roseiflexus |
| **bOTU95** | Armatimonadetes | uncultured |
| **bOTU107** | Acidobacteria | uncultured Acidobacteria bacterium |
| **bOTU93** | Proteobacteria | uncultured |
| **bOTU339** | Chloroflexi | uncultured |
| **bOTU313** | Gemmatimonadetes | uncultured |
| **bOTU46** | Nitrospirae | Nitrospira |
| **bOTU275** | Chloroflexi | uncultured |
| **bOTU63** | Proteobacteria | uncultured |
| **bOTU47** | Actinobacteria | Gaiella |
| **bOTU2961** | Actinobacteria | Agromyces |
| **bOTU89** | Chloroflexi | Roseiflexus |
| **bOTU244** | Actinobacteria | Blastococcus |
| **bOTU88** | Actinobacteria | uncultured |

The 50 most abundant bOTUs in the rhizosphere compartment are presented in the rank abundance plot below. The conditioning-dependent bOTUs among the Top50 rhizosphere bOTUs are marked with an asterisk. This graph was used for the Supplementary Figure 9.

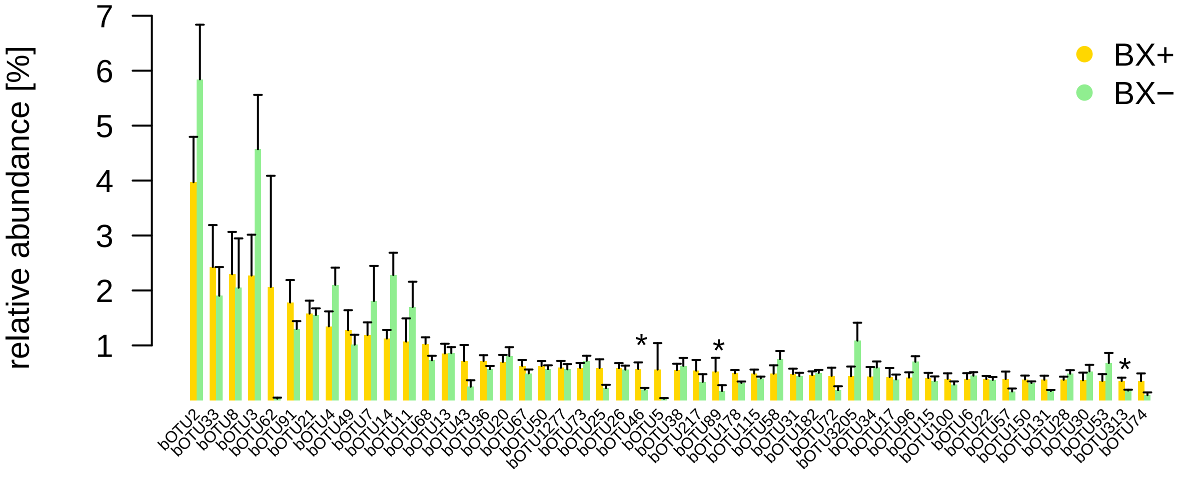


***Root samples: bOTUs differing between BX+ and BX- soils***

Below we list the direction and taxonomies of the differentially abundant bOTUs (between BX+ and BX- soils) in the root compartment. This information is documented in the Database S4.

| higher in BX+ | unchanged | higher in BX- |
|---|---|---|
| 8 | 159 | 17 |

| | phylum | genus |
|---|---|---|
| **bOTU133** | Actinobacteria | uncultured microorganism |
| **bOTU59** | Chloroflexi | Roseiflexus |
| **bOTU126** | Actinobacteria | uncultured Methylocystaceae bacterium |
| **bOTU1707** | Proteobacteria | uncultured |
| **bOTU185** | aquifer1 | uncultured |
| **bOTU339** | Chloroflexi | uncultured |
| **bOTU275** | Chloroflexi | uncultured |
| **bOTU47** | Actinobacteria | Gaiella |
| **bOTU168** | Proteobacteria | Janthinobacterium |
| **bOTU19** | Actinobacteria | Streptomyces |
| **bOTU5** | Deinococcus-Thermus | Deinococcus |
| **bOTU193** | Actinobacteria | unassigned |
| **bOTU2961** | Actinobacteria | Agromyces |
| **bOTU159** | Actinobacteria | Rubrobacter |
| **bOTU36** | Proteobacteria | unassigned |
| **bOTU62** | Proteobacteria | Pantoea |
| **bOTU88** | Actinobacteria | uncultured |
| **bOTU746** | Proteobacteria | Polaromonas |
| **bOTU1199** | Actinobacteria | unassigned |
| **bOTU225** | Actinobacteria | Gaiella |
| **bOTU244** | Actinobacteria | Blastococcus |
| **bOTU65** | Actinobacteria | Lapillicoccus |
| **bOTU51** | Chloroflexi | uncultured |
| **bOTU439** | Actinobacteria | Solirubrobacter |
| **bOTU21** | Proteobacteria | Variovorax |

The 50 most abundant bOTUs in the root compartment are presented in the rank abundance plot below. The conditioning-dependent bOTUs among the Top50 root bOTUs are marked with an asterisk. This graph was used for the Supplementary Figure 9.

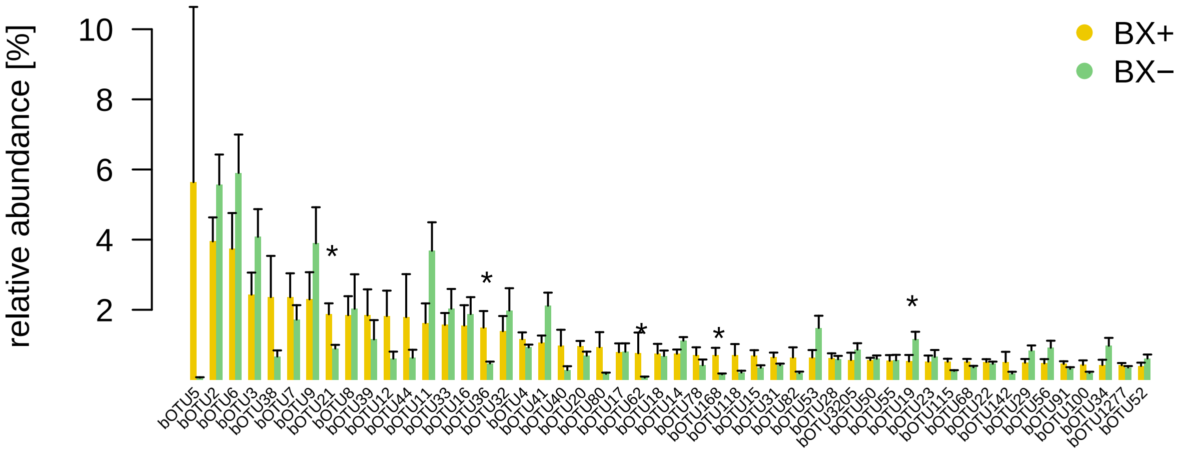


***soil conditioning-dependent bOTUs: rhizosphere vs. root compartments***

We then compared the differentially abundant bOTUs between BX+ and BX- soils of the rhizosphere compartment with the ones from the root samples. We found that half of the rhizosphere bOTUs that were senstive to the previous soil conditioning were also differentially abundant in the root compartment. More bOTUs respond to the previous soil conditioning in the root compartment (Venn diagram below, this plot was incorporated into the Supplementary Figure 9).

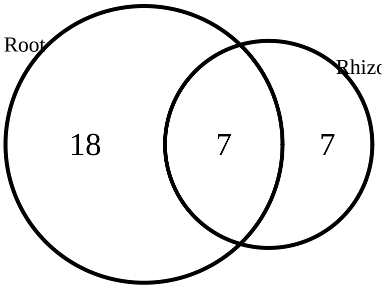


Conclusion: bacterial communities reveal marked patterns in response to soil conditioning.
