# Supplementary material for: Root exudate metabolites drive plant-soil feedbacks on growth and defense by shaping the rhizosphere microbiota
Source: Nat Commun. 2018 Jul 16;9:2738. doi: 10.1038/s41467-018-05122-7 (PMC6048113; doi:10.1038/s41467-018-05122-7)
Supplement: Supplementary file 7 — Supplementary Data 3 [file 41467_2018_5122_MOESM7_ESM.zip › Supplementary Data 3.zip!/Supplementary Data 3/Supplementary Data 3/Hu_et_al_fungi_community_analysis_in_R.pdf]

Hu et al. (2018), fungi community analysis in R

Klaus Schlaeppi

2018-04-18

Contents

Background **2**

Overview all data **2**

Sequencing effort . . . 2
Community similarities between field and feedback experiments . . . 3

Analysis field experiment **4**

Alpha diversity . . . 4
Beta diverstiy . . . 5
PCoA - unconstrained ordination . . . 5
CAP - constrained ordination . . . 5
PERMANOVA . . . 6
Identification of differentially abundant fOTUs . . . 8

Analysis feedback experiment **11**

Alpha diversity . . . 11
Beta diverstiy . . . 12
PCoA - unconstrained ordination . . . 12
Constrained ordination . . . 12
PERMANOVA . . . 15
Identification of differentially abundant fOTUs . . . 16

Background

We have sequenced the fungi communities of a field experiment and a feedback experiment. We have prepared one library with samples from both experiments and sequenced them in a single sequencing run and the raw data is stored in the same ENA archive as one of the 16S datasets. The raw data of the ITS sequencing run were processed in the same way as the 16S data (bioinformatic script available in Database S2). The high quality sequences were combined for inferring fungal OTUs (fOTUs) and generation of an fOTU table (Database S2). The analysis of fungal communities in R requires the following input files:
- Experimental design: *Database_S1.txt* (save Database S1 as tab-delimited text file)
- OTU table: *its_all_samples_trimmed_qfiltered_renamed_derep_ab5_otu.tab*
- Taxonomy: *UNITE_tax_forR*

Overview all data

Sequencing effort

For the field experiment we generated a total of 1'431'681 high quality sequences with a median sequence number of 33'352 sequences per sample (range: 17'473 to 45'971). The feedback experiment yielded a total of 1'347'548 high quality sequences with a median sequence number of 34'036 sequences per sample (range: 17'089 to 50'420).

The field and feedback experiments comprise the following sample groups:

Field: 5 groups with soil samples; rhizosphere and root samples of both wildtype B73 or *bx1* mutant plants.

Feedback: 4 groups with rhizosphere and root samples of wildtype B73 plants that were grown both on B73 (BX+) or *bx1* (BX-) conditioned soils.

The mean sequence numbers per sample group are shown in the plot below.

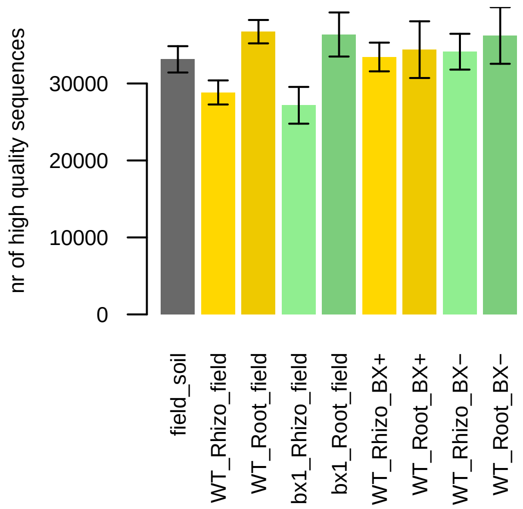

To decide on how to normalize the data we followed the recommendation of Weiss et al. (2015, PeerJ) and we inspected whether there are differences in sequencing depths between the different sample groups utilizing the non-parametric Kruskal-Wallis Test.

```
##
##  Asymptotic Kruskal-Wallis Test
##
## data:  colSums(fDAT) by
##   design$groups (field_soil, WT_Rhizo_field, WT_Root_field, bx1_Rhizo_field, bx1_Root_field, WT_Rhizo
## chi-squared = 13.749, df = 8, p-value = 0.08854
```

Although, we did not find significant differences in sequence numbers between the sample groups, we still decided to employ rarefication for normalization of the fungal data because we wanted to treat the ITS data the same way as for the 16S data (where we found significant differences in sequence numbers between the sample groups). Rarefication disrupts most reliably possible effects between sample groups that are due to sampling depth (see Weiss et al., 2015 PeerJ).

Community similarities between field and feedback experiments

Please note: the setups of field and feedback experiments differ substantially. The field experiment used ***full field soil*** and ***natural conditions*** while the feedback experiment utilized ***sand-amended field soil in pots*** and ***controlled greenhouse conditions***. These experimental factors are likely to confound such a comparison. Only for curiosity, we inspect here the community similarities between both experiments using the r-package ‘phyloseq’ and as input, we employed rarefied data (rarefied to the minimal sampling depth of both experiments, 17’089 sequences per sample). The plot below consists of an Principal Coordiante Analysis (PCoA) with Bray-Curtis distances utilizing the data of both the field and the feedback experiments and shows that the root and rhizosphere compartments differ among themselves and as suspected also between the two experiments. Because of confounding experimental factors we renounced comparing the field with the feedback experiment for the analysis that we present in the manuscript.

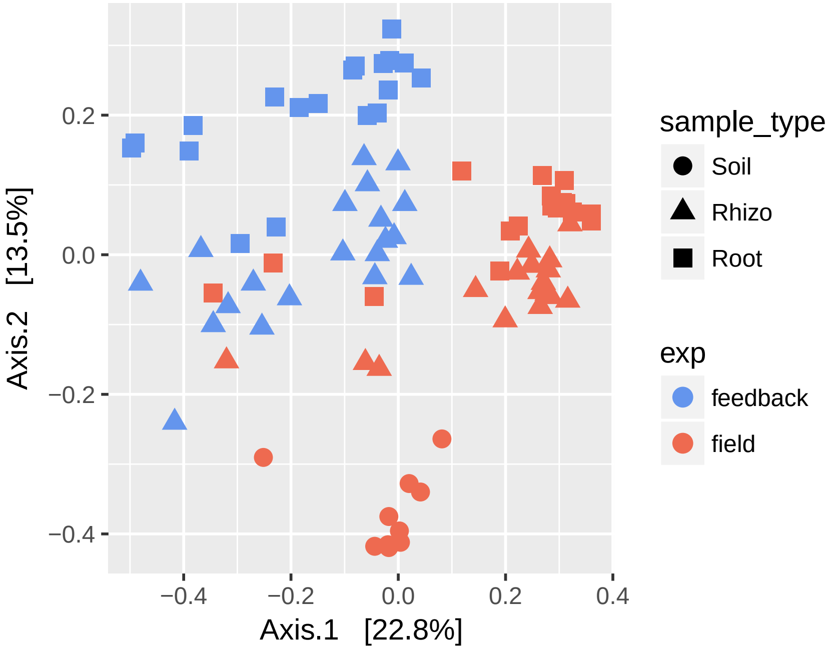

Analysis field experiment

The field experiment consistet of a total of 44 samples representing 5 sample groups (soil samples and rhizosphere and root samples of both wildtype B73 or *bx1* mutant plants). The number of replicates per sample group is given below.

field_soil	WT_Rhizo	WT_Root	bx1_Rhizo	bx1_Root
10	10	10	7	7

Alpha diversity

We examined alpha (within sample) diversity patterns of the field experiment data using the indices of Chao's estimate of richness, Shannon diversity and Pielou's evenness indices. We were interested in alpha diversity patterns between sample types and if BX exudation would affect microbial diversity. We utilized the 'estimate_richness' function of the r-package 'phyloseq' and employed rarefied data (rarefied to the minimal sampling depth in the field experiment, 17'473 sequences per sample) as input. The plots below report Chao-1 richness, Shannon diversity and Pielou's evenness.

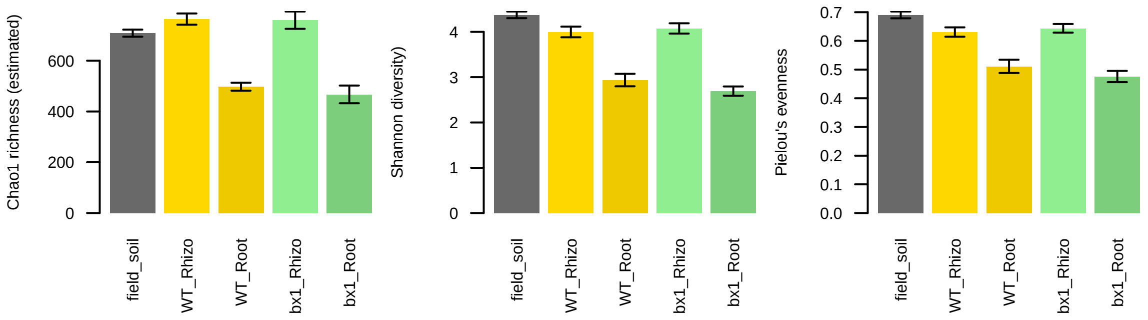

Statistic examination using Analysis of Variance (ANOVA, model with ~ *sample type / plant genotype*) yielded significant differences between *sample type* (soil, rhizosphere and root) for Chao-1 richness P=4.88e-21, Shannon diversity P=9.84e-13 and Pielou's evenness P=1.53e-11. Althought, all three mean diversity indices tend to be slightly lower in *bx1*(B73) compared to B73 (both in roots and rhizosphere samples), the F-tests for the factor *plant genotype* (B73 or *bx1*(B73)) were *not* significant (Chao-1 richness P=5.53e-01, Shannon diversity P=5.72e-01 and Pielou's evenness P=3.63e-01). Therefore, no further examination with a post-hoc test was conducted.

Beta diverstiy

PCoA - unconstrained ordination

In a first step, we examined the field experiment data for beta diversity patterns. In particular, we were interested in community similarites between the sample groups. We utilized the r-package 'phyloseq' and employed rarefied data (rarefied to the minimal sampling depth in the field experiment, 17'473 sequences per sample) as input. The plot below reports the PCoA (unconstrained ordination) based on Bray Curtis distances (Supplementary Figure 1).

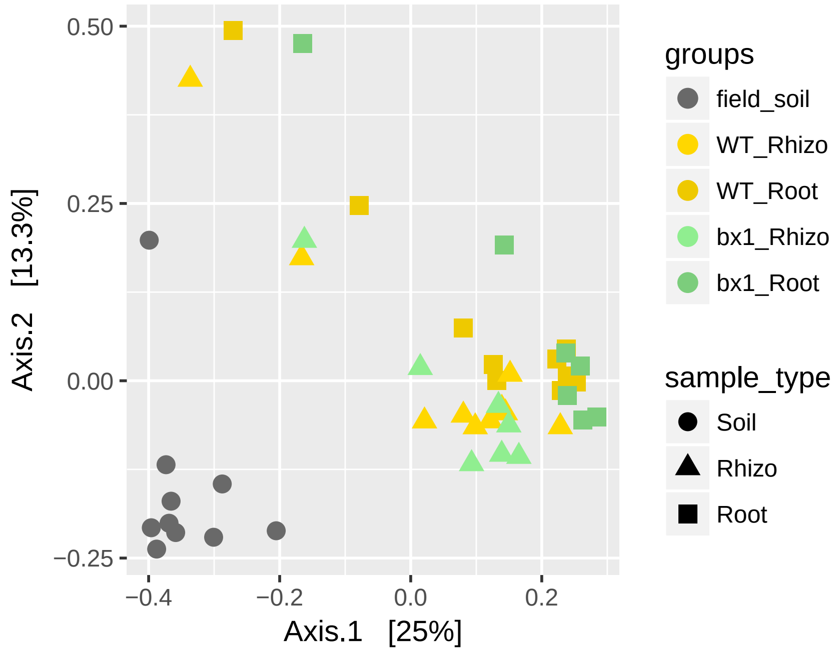

CAP - constrained ordination

We then used constrained ordination (partial canonical analysis of principal coordinates; CAP) to evaluate how the variation in the dataset can be partitioned to the experimental factors *sample type* (soil, rhizosphere and root) and *plant genotype* (B73 or *bx1*(B73)). A model with nesting *plant genotype* within the factor *sample type* was used for the CAP (~*sample type/plant genotype*). We validated the significance of the model with ANOVA using 99'999 permutations (table below). The plot below reports the CAP based on Bray Curtis distances (Figure 1 in the manuscript) with the title stating the constrained factor(s), their amount of explained variation and their statistic significance.

	Df	SumOfSqs	F	Pr(>F)
**Model**	4	3.096	4.637	1e-05
**Residual**	39	6.51	NA	NA

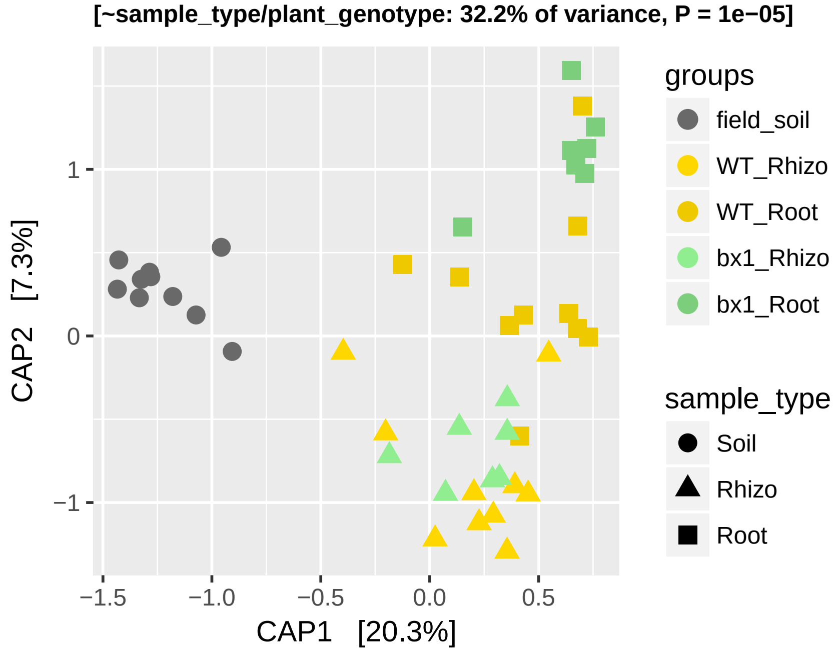

Conclusions: Both PCoA and CAP reveal that the different compartments have specific compositions in their fungal communities. In addition, the exudation of BXs appears to have a stronger effect on the root compared to rhizosphere communities.

PERMANOVA

We utilized Permutational Multivariate Analysis of Variance (PERMANOVA) to partition and quantify the effects of the experimental factors *sample type* (soil, rhizosphere and root) and *plant genotype* (B73 or *bx1* (B73)). For this we utilized the function 'adonis' of the r-package 'vegan' with a Bray Curtis distance matrix as input and running 99'999 permutations. Again, we fitted a model with nesting *plant genotype* within the factor *sample type* (*~sample type/plant genotype*). The PERMANOVA output below is reported as Supplementary Table 1.

Table 3: Table continues below

	Df	SumsOfSqs	MeanSqs	F.Model	R2
**sample_type**	2	2.603	1.301	7.797	0.271
**sample_type:plant_genotype**	2	0.4928	0.2464	1.476	0.0513
**Residuals**	39	6.51	0.1669	NA	0.6777
**Total**	43	9.606	NA	NA	1

	Pr(>F)
**sample_type**	1e-05
**sample_type:plant_genotype**	0.07109
**Residuals**	NA
**Total**	NA

We also conducted pairwise comparison between the five sample groups (soil, WT rhizosphere, bx1 rhizoshpere, WT root and bx1 root samples) utilizing the function 'pairwise.perm.manova' of the r-package 'RVAideMemoire'

with the same Bray Curtis distance matrix as input and running 99'999 permutations. We fitted a simple model with the five sample groups as factor. The pairwise PERMANOVA output below is reported in Supplementary Table 2. The reported *P*-values are adjusted for false discovery rate (fdr).

	field_soil	WT_Rhizo	WT_Root	bx1_Rhizo
**WT_Rhizo**	0.00015	NA	NA	NA
**WT_Root**	1e-04	0.04761	NA	NA
**bx1_Rhizo**	0.00016	0.251	0.002057	NA
**bx1_Root**	0.00016	0.00016	0.07068	0.00165

Differences in beta-diversity can be driven by true biological differences between groups, or differences in group dispersion (variance), or both (Anderson MJ. A new method for non parametric multivariate analysis of variance. Austral Ecol. 2001;26:32–46.). Therefore, we tested for differences in dispersion between the five sample groups (soil, WT rhizosphere, bx1 rhizoshpere, WT root and bx1 root samples) using the functions 'betadisper' and 'permutest' of the r-package 'vegan'. The pairwise BETADISP output below is reported in Supplementary Table 2. Lack of significance in the dispersion tests supports that differences between the sample groups are driven by true biological differences and do not present an artifact due to differences in dispersion.

	observed	permuted
**field_soil-WT_Rhizo**	0.2423	0.2451
**field_soil-WT_Root**	0.8001	0.8034
**field_soil-bx1_Rhizo**	0.0376	0.03404
**field_soil-bx1_Root**	0.1353	0.135
**WT_Rhizo-WT_Root**	0.2617	0.2667
**WT_Rhizo-bx1_Rhizo**	0.7444	0.7491
**WT_Rhizo-bx1_Root**	0.6241	0.6291
**WT_Root-bx1_Rhizo**	0.1291	0.1294
**WT_Root-bx1_Root**	0.1707	0.172
**bx1_Rhizo-bx1_Root**	0.7992	0.8046

In conclusion: the pairwise PERMANOVA revealed that fungal communities differ significantly between the *sample types* (soil, rhizosphere, and root samples), while the communities differed marginally between *plant genotype* in roots but not the rhizosphere. The pairwise BETADISP tests supported (significant differences between the sample groups are driven by true biological differences and not due to differences in dispersion) most of these comparisons. The comparisons between soil and bx1 rhizosphere samples presented an exception because the differences arise from differences in dispersion of the data and therefore, cannot be considered significantly different from each other.

Identification of differentially abundant fOTUs

In a second step, we identified both in rhizosphere and root samples fOTUs that differed in their relative abundance between wildtype B73 and mutant *bx1* (B73) plants. For this analysis we focussed on the abundant OTUs similar to Bai et al. (2016, Nature). We defined abundant fOTUs as fOTUs that have a minimal average abundance of 0.1% in the dataset. We utilized the r-package 'edgeR' for this analysis as particular strengths of this tool are the ability to estimate biological dispersion between replicate samples, and the performance of exact tests of significance that are suitable for small counts. We estimated dispersion by weighted likelihood empirical Bayes, modeled the OTU abundances as a function for the five sample groups and we contrasted the two genotypes, both for rhizosphere and root samples for the likelyhood ratio tests. P-values were adjusted according to Benjamini and Hochberg (1995).

Of the 1'861 fOTUs in the field dataset, 118 had a higher minimal average abundance than 0.1%.

Rhizosphere samples: fOTUs differing between B73 and **bx1** *plants*

Below we list the direction and taxonomies of the differentially abundant fOTUs (between B73 and *bx1* plants) in the rhizosphere compartment. This information is documented in the Database S4.

lower in WT	unchanged	higher in WT
2	115	1

	phylum	class
**fOTU51**	unassigned	unassigned
**fOTU98**	Ascomycota	unassigned
**fOTU54**	Ascomycota	Sordariomycetes

The 50 most abundant fOTUs in the rhizosphere compartment are presented in the rank abundance plot below. The BX-dependent fOTUs among the Top50 rhizosphere fOTUs are marked with an asterisk. This graph was used for the Supplementary Figure 1.

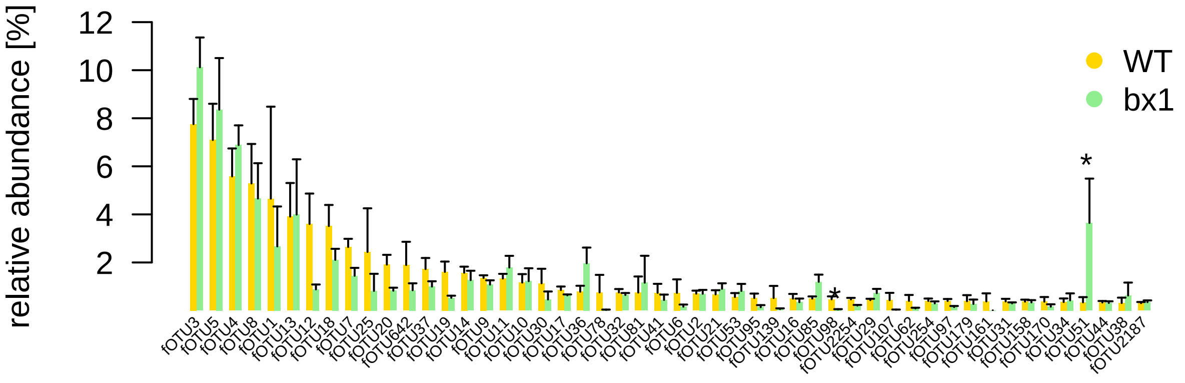

Root samples: fOTUs differing between B73 and* bx1 *plants

Below we list the direction and taxonomies of the differentially abundant fOTUs (between B73 and *bx1* plants) in the root compartment. This information is comprised in the Database S4.

lower in WT	unchanged	higher in WT
6	108	4

	phylum	genus
**fOTU21**	Ascomycota	Fusarium
**fOTU57**	Ascomycota	Fusarium
**fOTU36**	Ascomycota	Cylindrocarpon
**fOTU66**	Ascomycota	unassigned
**fOTU35**	Ascomycota	unassigned
**fOTU7**	Ascomycota	Periconia
**fOTU4**	Ascomycota	Fusarium
**fOTU98**	Ascomycota	unassigned
**fOTU59**	Ascomycota	Pseudeurotium
**fOTU20**	Glomeromycota	Funneliformis

The 50 most abundant fOTUs in the root compartment are presented in the rank abundance plot below. The BX-dependent fOTUs among the Top50 root fOTUs are marked with an asterisk. This graph was used for the Supplementary Figure 1.

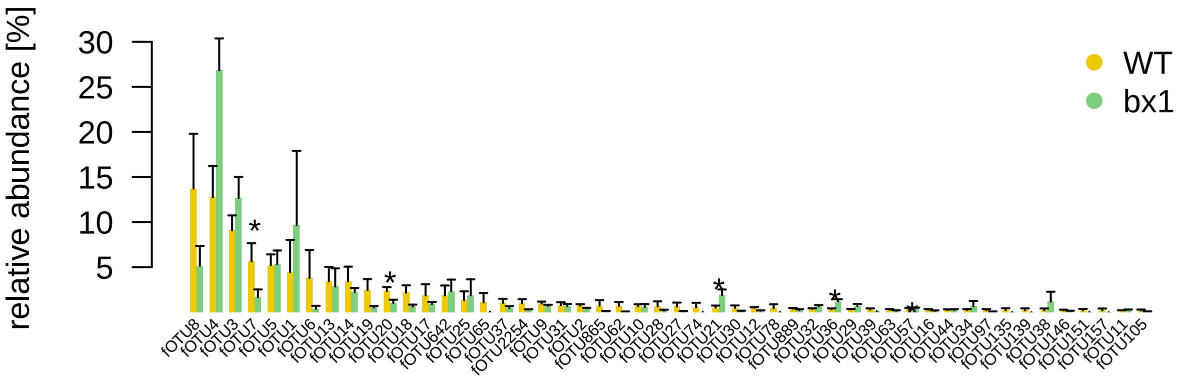

BX-dependent fOTUs: rhizosphere vs. root compartments

We then compared the differentially abundant fOTUs between B73 and *bx1* plants of the rhizosphere compartment with the ones from the root samples. We found that most fOTUs were senstive to the exudation of BX in a compartment-specific manner (Venn diagram below, this plot was incorporated into the Supplementary Figure 1).

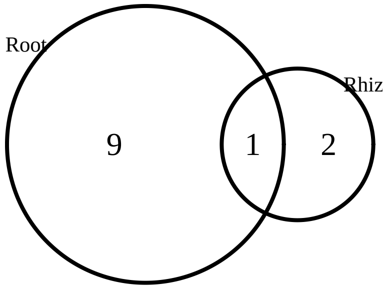

The bargraphs below reports the fOTU98 of the intersection that was BX-dependent in both compartments. Additionally, the Funneliformis fOTU20 is shown.

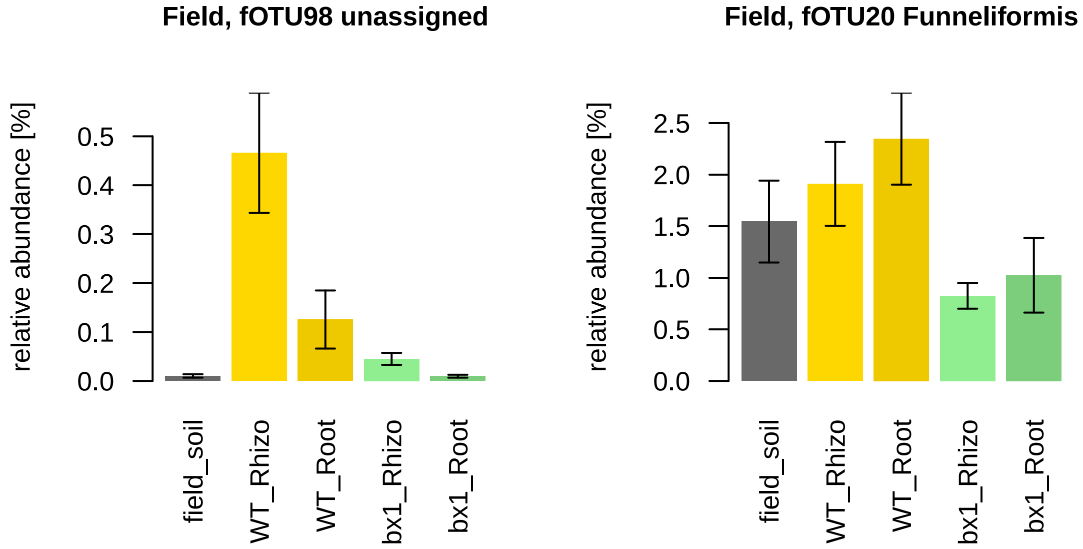

Analysis feedback experiment

Note: we followed the same logic for the analysis of the feedback experiment and we utilized the same r-packages and code as we did for the field experiment.

The feedback experiment consistet of a total of 39 samples representing 4 sample groups (rhizosphere and root samples of wildtype B73 plants that were grown both on B73 (BX+) or *bx1* (BX-) conditioned soils). One sample ("Root_18") was removed due to low sequence coverage. The number of replicates per sample group is given below.

Rhizo_WT_BX+	Root_WT_BX+	Rhizo_WT_BX-	Root_WT_BX-
10	9	10	10

Alpha diversity

We examined alpha (within sample) diversity patterns of the feedback experiment data using the indices of Chao's estimate of richness, Shannon diversity and Pielou's evenness. We were interested in alpha diversity patterns between sample types and if BX conditioning would affect microbial diversity. We utilized the 'estimate_richness' function of the r-package 'phyloseq' and employed rarefied data (rarefied to the minimal sampling depth in the feedback experiment, 17'089 sequences per sample) as input. The plots below report Chao-1 richness, Shannon diversity and Pielou's evenness.

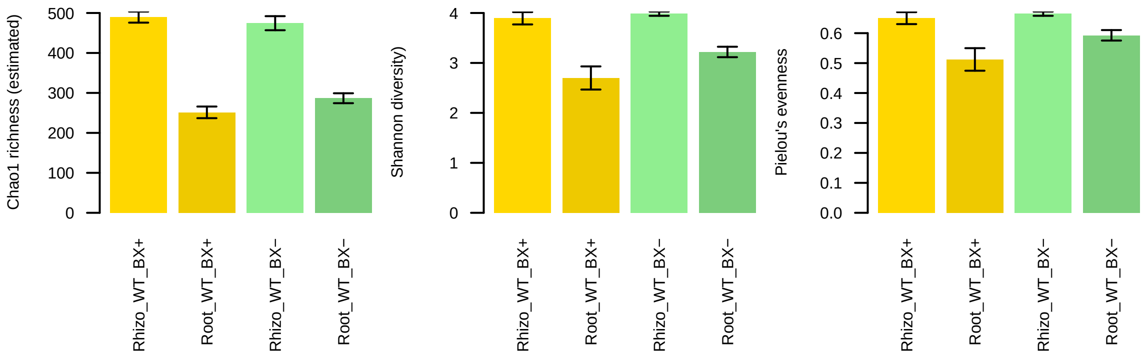

Statistic examination using Analysis of Variance (ANOVA, model with ~ *sample type* x *soil conditioning*) yielded significant differences between *sample type* (rhizosphere and root) for Chao-1 richness *P*=1.47e-17, Shannon diversity *P*=1.92e-09 and Pielou's evenness *P*=5.18e-05. Chao-1 richness *P*=1.06e-01 and Shannon diversity *P*=1.3e-01 were unaffected by *soil conditioning* (BX+ or BX-; from previous plants B73 or *bx1*, respectively). We noted a weak difference in Pielou's evenness *P*=4.35e-02 due to the type of *soil conditioning*. The interaction term did not reveal significant differences (Chao-1 richness *P*=1.3e-01, Shannon diversity *P*=4.52e-01 and Pielou's evenness *P*=1.5e-01).

Beta diverstiy

PCoA - unconstrained ordination

We first examined the feedback experiment data for beta diversity patterns between the sample groups. The plot below was used for Supplementary Figure 9 and reports the PCoA (unconstrained ordination) based on Bray Curtis distances (Input: rarefied data at sampling depth 17'089 sequences per sample).

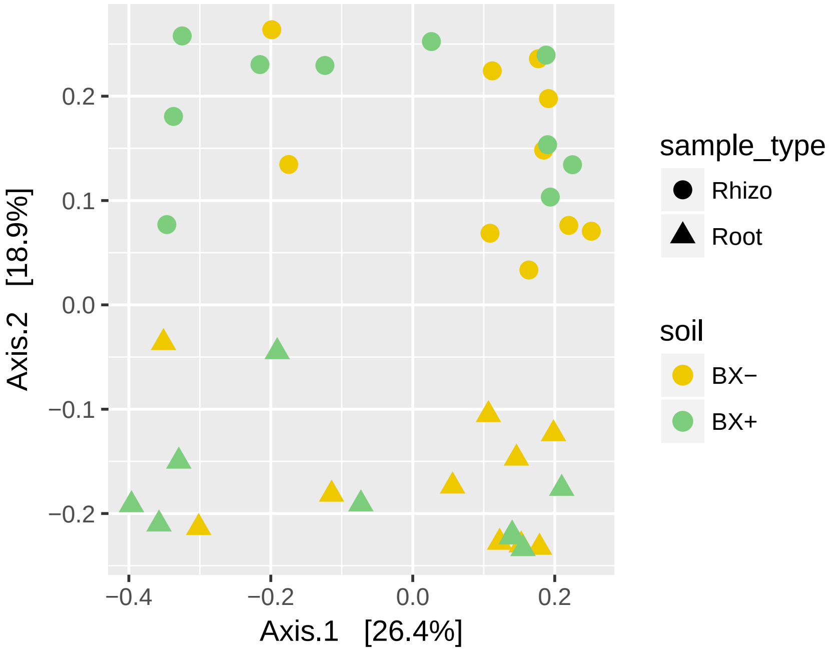

Conclusion: We confirm that root and rhizosphere microbioms are dissimilar (PCo Axis 2). Within each sample type, the type of previous soil conditioning (BX+ or BX-) appears to separate the fungal communities (PCo Axis 1).

Constrained ordination

We then also conducted a CAP analysis to evaluate how the variation in the dataset can be partitioned to the experimental factors *sample type* (rhizosphere and root) and *soil conditioning* (BX+ or BX-). We fitted the same model as with PERMANOVA (~ *sample type* * *soil conditioning*) and validated its significance with ANOVA using 99'999 permutations (table below).

	Df	SumOfSqs	F	Pr(>F)
**Model**	3	1.611	3.61	1e-05
**Residual**	35	5.206	NA	NA

For the feedback experiment, we explored in addition to the sample scores also the fOTU scores of the CAP. The fOTU scores in such a bi-plot analysis present orthogonal projections for each fOTU that fit the sample distribution in the ordination space. In simple terms, the bi-plot analysis reveals fOTUs that contribute to explain the sample distribution pattern in the constrained ordiantion. We did not perform a bi-plot analysis for the field experiment data, as there the occurrence of soil samples obscures meaningful interpretations with respect to *plant geotype*.

Here, we subsetted the OTU table to fOTUs that belong to the 6 most abundant phyla (this was done as preparation for the subsequent bi-plot analysis; otherwise the bi-plot becomes unclear with OTUs of too

many phyla). The sample scores plot based on fOTUs of 6 most abundant Phyla differes only marginally compared to a plot with OTUs of all phyla in the dataset. The plot below reports the sample scores of the CAP based on Bray Curtis distances and is presented as Figure 7 in the manuscript. The plot title states the constrained factor(s), their amount of explained variation and their statistic significance.

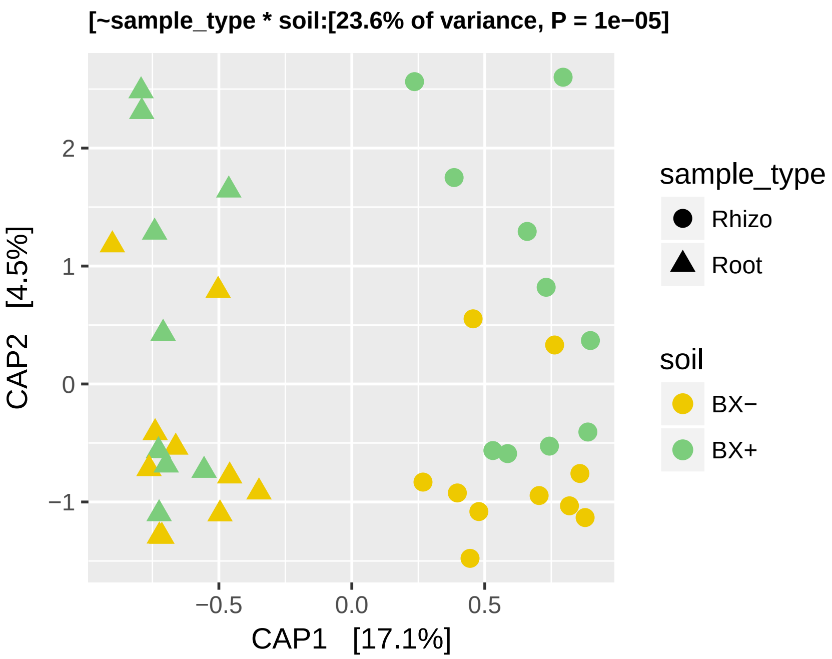

Below is the plot with the fOTU scores of the CAP (presented in Figure 7). Please note, the coordiantes of the sample and the OTU plot are congruent. We defined “explanatory” OTUs as fOTUs based on their position along CAP axes 1 (< -0.1 or > 0.1) and CAP axis 2 (< -0.1 or > 0.1). “Explanatory” OTUs are indicated in the bi-plot with their OTU ID.

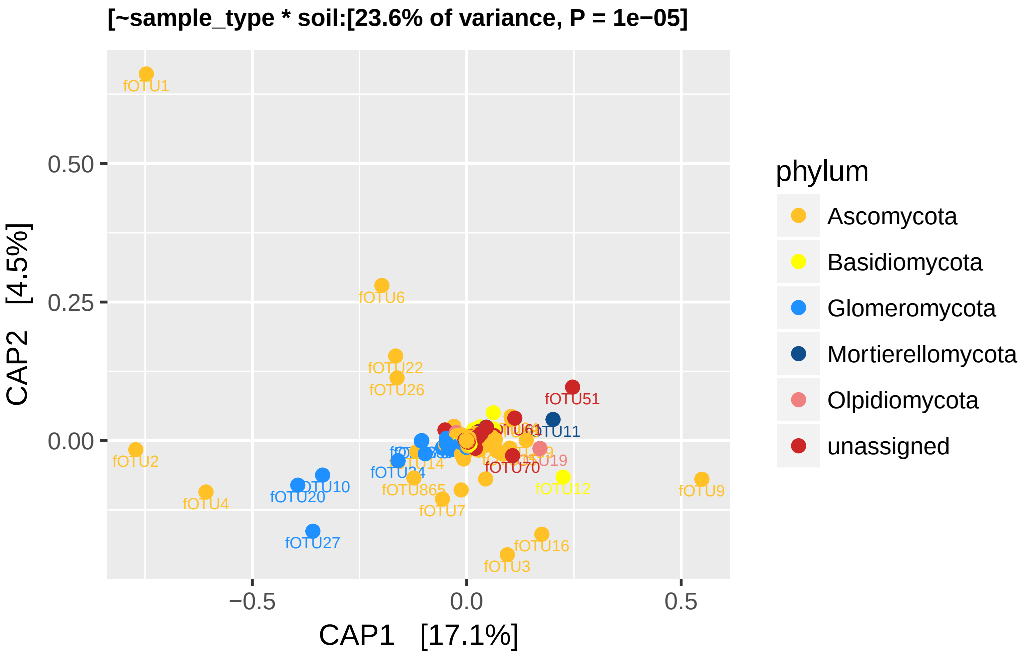

Conclusions: Subsets of Ascomycetes and Glomeromycetes discriminate mainly the fungal communities of rhizosphere and root compartments (along CAP axis 1). In roots, Glomeromycetes tend to be higher in abundance in BX- conditioned soils while some Ascomycetes are more abundant in BX+ conditioned soils.

The bargraphs below present a few "explanatory" OTUs of the CAP and they illustrate how to read bi-plot graphs. One example for each cardinal point of the ordination space was chosen.

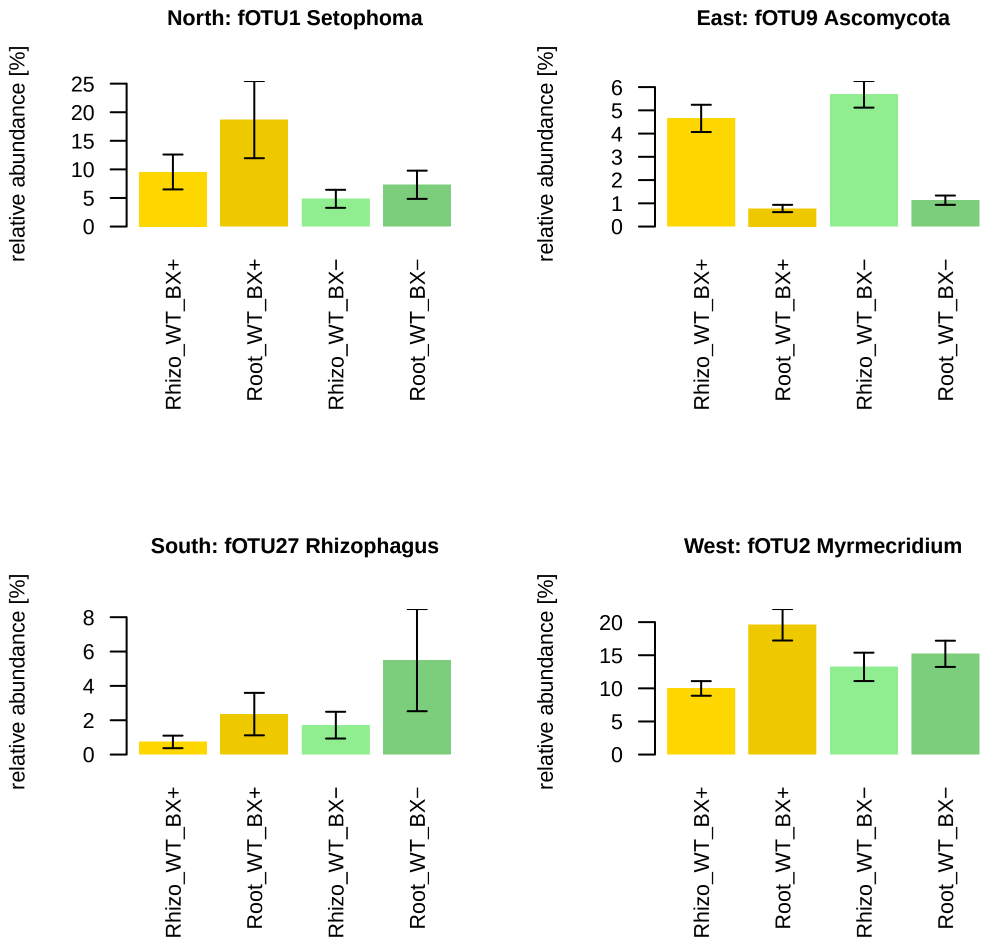

PERMANOVA

PERMANOVA was used to partition and quantify the effects of the experimental factors *sample type* (rhizosphere and root) and *soil conditioning* (BX+ or BX-; from previous plants B73 or *bx1*, respectively). A Bray Curtis distance matrix was used as input, we runned 99'999 permutations and fitted a model with interaction between the factors *sample type* and *soil conditioning* (~ *sample type* * *soil conditioning*). The PERMANOVA output below is reported as Supplementary Table 7.

	Df	SumsOfSqs	MeanSqs	F.Model	R2	Pr(>F)
**sample__type**	1	1.193	1.193	7.974	0.174	1e-05
**soil**	1	0.3098	0.3098	2.07	0.04518	0.03616
**sample__type:soil**	1	0.1158	0.1158	0.7739	0.01689	0.6338
**Residuals**	35	5.238	0.1497	NA	0.7639	NA
**Total**	38	6.857	NA	NA	1	NA

We also conducted pairwise comparison between the sample groups (rhizosphere and root, with and without BX conditioning) utilizing the function 'pairwise.perm.manova' of the r-package 'RVAideMemoire' with the same Bray Curtis distance matrix as input and running 99'999 permutations. We fitted a simple model with the four sample groups as factor. The pairwise PERMANOVA output below is reported in Supplementary Table 8. The reported *P*-values are adjusted for false discovery rate (fdr).

	Rhizo__WT__BX+	Root__WT__BX+	Rhizo__WT__BX-
**Root__WT__BX+**	0.001695	NA	NA
**Rhizo__WT__BX-**	0.08596	6e-05	NA
**Root__WT__BX-**	0.00014	0.3212	0.00014

Differences in beta-diversity can be driven by true biological differences between groups, or differences in group dispersion (variance), or both (Anderson MJ. A new method for non parametric multivariate analysis of variance. Austral Ecol. 2001;26:32–46.). Therefore, we tested for differences in dispersion between the sample groups using the functions 'betadisper' and 'permutest' of the r-package 'vegan'. The pairwise BETADISP output below is reported in Supplementary Table 8. Lack of significance in the dispersion tests supports that differences between the sample groups are driven by true biological differences and do not present an artifact due to differences in dispersion.

	observed	permuted
**Rhizo__WT__BX+-Root__WT__BX+**	0.9316	0.9307
**Rhizo__WT__BX+-Rhizo__WT__BX-**	0.02368	0.02333
**Rhizo__WT__BX+-Root__WT__BX-**	0.3072	0.3107
**Root__WT__BX+-Rhizo__WT__BX-**	0.05405	0.05321
**Root__WT__BX+-Root__WT__BX-**	0.3629	0.3655
**Rhizo__WT__BX–Root__WT__BX-**	0.2755	0.2749

In conclusion: While PERMANOVA identified significant effects by the experimental factors *sample type* (rhizosphere and root) and a weak effect by *soil conditioning* (BX+ or BX-), this was not confirmed by the pairwise tests, where only the comparison between sample types were significant. The pairwise BETADISP tests supported most of these comparisons and additionally identified differences in dispersion between BX+ and BX- conditioned rhizosphere samples.

Identification of differentially abundant fOTUs

In a second step, we identified both in rhizosphere and root samples fOTUs that differed in their relative abundance between the conditioned soils *BX+* (B73 as previous plant genotype) and *BX-* (mutant *bx1* as previous plant). Analogous to the above, we focussed on the abundant fOTUs, we utilized 'edgeR', we modeled the fOTU abundances as a function for the four sample groups and we inspected the contrasts between the two genotypes both in rhizosphere and root samples. P-values were adjusted according to Benjamini and Hochberg (1995).

Of the 1'741 fOTUs in the feedback dataset, 102 had a higher minimal average abundance than 0.1%.

Rhizosphere samples: fOTUs differing between BX+ and BX- soils

Below we list the direction and taxonomies of the differentially abundant fOTUs (between BX+ and BX- soils) in the rhizosphere compartment. This information is documented in the Database S4.

higher in BX+	unchanged	higher in BX-
0	102	0

The 50 most abundant fOTUs in the rhizosphere compartment are presented in the rank abundance plot below. The conditioning-dependent fOTUs among the Top50 rhizosphere fOTUs are marked with an asterisk. This graph was used for the Supplementary Figure 9.

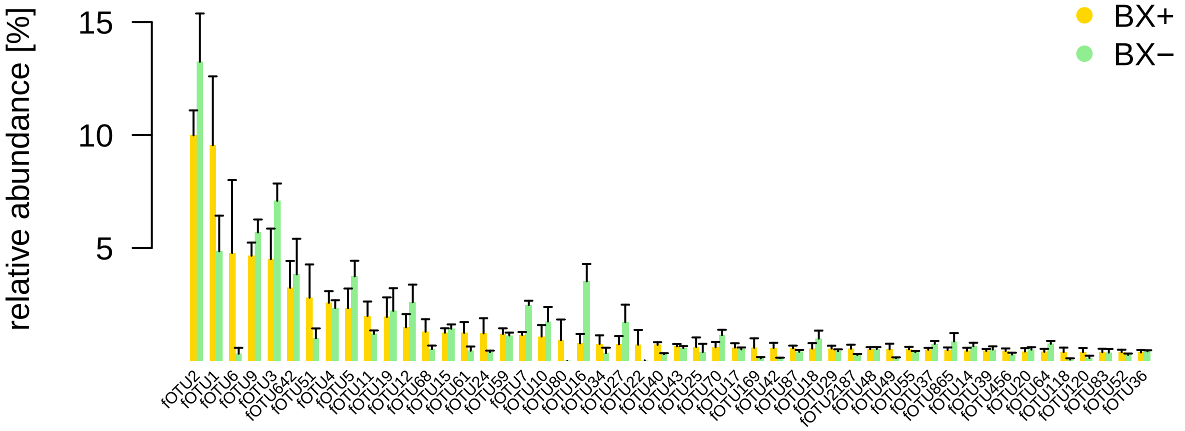

Root samples: fOTUs differing between BX+ and BX- soils

Below we list the direction and taxonomies of the differentially abundant fOTUs (between BX+ and BX- soils) in the root compartment. This information is documented in the Database S4.

higher in BX+	unchanged	higher in BX-
0	101	1

	phylum	genus
**fOTU58**	Basidiomycota	Cystofilobasidium

The 50 most abundant fOTUs in the root compartment are presented in the rank abundance plot below. The conditioning-dependent fOTUs among the Top50 root fOTUs are marked with an asterisk. This graph was used for the Figure 9.

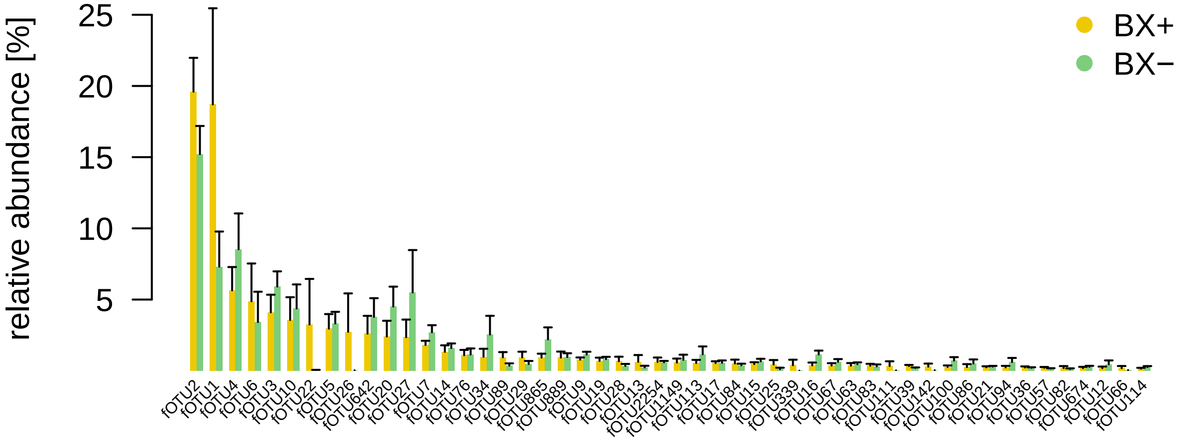

soil conditioning-dependent fOTUs: rhizosphere vs. root compartments

We then compared the differentially abundant fOTUs between BX+ and BX- soils of the rhizosphere compartment with the ones from the root samples. We found that half of the rhizosphere fOTUs that were senstive to the previous soil conditioning were also differentially abundant in the root compartment. More OTUs respond to the previous soil conditioning in the root compartment (Venn diagram below, this plot was incorporated into the Supplementary Figure 9).

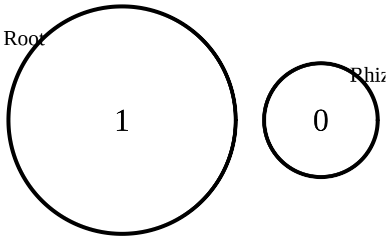

Conclusion: fungal communities were more variable than bacterial communities. No marked fungal pattern in response to soil conditioning could be identified.
